# Iridium-Catalyzed Regio- and Diastereoselective Synthesis of C-Substituted Piperazines

**DOI:** 10.1021/acscatal.2c05895

**Published:** 2023-02-16

**Authors:** Luis Tarifa, M. Pilar del Río, Laura Asensio, José A. López, Miguel A. Ciriano, Ana M. Geer, Cristina Tejel

**Affiliations:** Departamento de Química Inorgánica, Instituto de Síntesis Química y Catálisis Homogénea (ISQCH), CSIC-Universidad de Zaragoza, Pedro Cerbuna 12, 50009 Zaragoza, Spain.

**Keywords:** piperazines, iridium, homogeneous catalysis, [3 + 3]-cycloadditions, imines, trimethylamine *N*-oxide

## Abstract

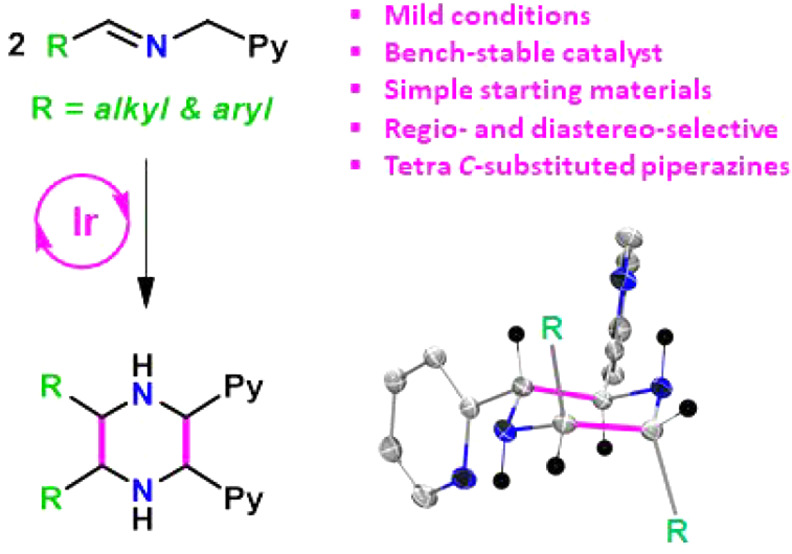

Piperazine rings are essential motifs frequently found in commercial drugs. However, synthetic methodologies are mainly limited to *N*-substituted piperazines, preventing structural diversity. Disclosed herein is a straightforward catalytic method for the synthesis of complex C-substituted piperazines based on an uncommon head-to-head coupling of easily prepared imines. This 100% atom-economic process allows the selective formation of a sole diastereoisomer, a broad substrate scope, and a good functional group tolerance employing a bench-stable iridium catalyst under mild reaction conditions. Key to the success is the addition of *N*-oxides to the reaction mixture, as they notably enhance the catalytic activity and selectivity.

The piperazine ring is a key pharmacophore for a wide range of drugs, including those with antibiotic, antidepressant, anti-HIV, anticancer, antiviral, antimicrobial, and anxiolytic activities (some examples are shown in Figure 1).^1^ Considerable efforts have been devoted to the development of synthetic routes yielding this privileged drug scaffold, which have traditionally focused on the reduction of diketopiperazines, the reductive amination of dicarbonyl compounds, and transition-metal-catalyzed cyclization reactions.^2^ These methods often require multistep synthesis, as well as protecting and deprotecting steps.^3^ More sustainable approaches include a “borrowing hydrogen” method, which uses 1,5-diols and primary amines, the synthesis of 2-substituted piperazines by an iridium photocatalyst,^4^ and biocatalytic reductive aminations of 1,2-dicarbonyl and 1,2-diamine substrates, which yield piperazines in an atom-economical fashion.^5^

**Figure 1:**
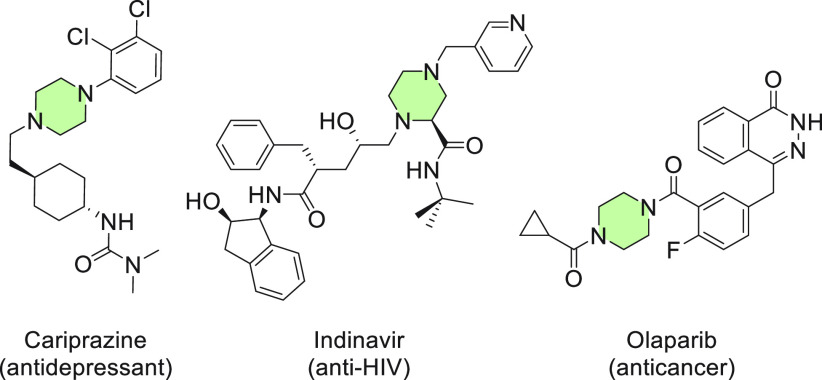
Selected examples of pharmaceuticals containing a piperazine motif.

As for any drug, the individual architecture is crucial for the drug–target interactions and therefore directly impacts on the inherent properties and specific function of the resulting molecule.^1e^ Moreover, the increase of molecular complexity and the number of stereogenic centers, also referred to as escaping from flatland, has been deemed key for the exploration of chemical space potentially, leading to unexplored molecular recognition with biological receptors within an active site.^6^ These altered vectors can be advantageous, leading to chemical diversity and unique pharmaceutical activities.

An analysis of piperazine cores in pharmaceuticals reveals limited structural diversity, with most examples containing substituents on the N atoms, but limited examples of C-substituted piperazines.^1a,1c,1d^ Whereas the functionalization of the nitrogen atoms is relatively straightforward, the postsynthetic functionalization of the carbon atoms can be extremely challenging.^7^ Therefore, there is great interest in the development of new synthetic routes yielding carbon-substituted piperazines in a straightforward manner.^1b^

In this context, easily prepared imines featuring the “CH=N—CH_2_” motif could be valuable synthons to C-substituted piperazines via the dimerization of the highly reactive azomethine ylide isomer (Scheme 1).

**Scheme 1:**
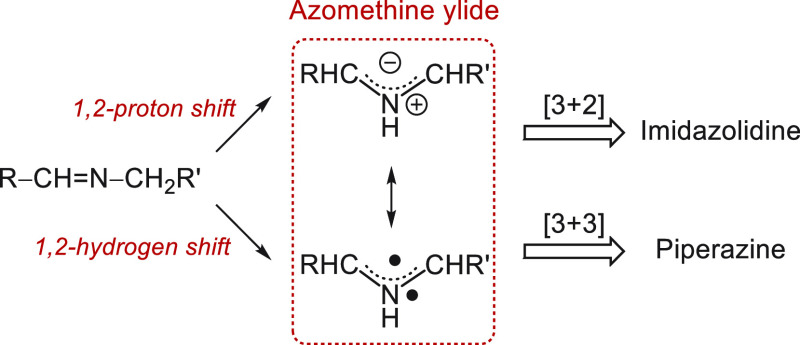
Pathways for Azomethine Ylides from Imines, Zwitterion and Diradical Resonant Forms, and Possible Cycloaddition Reactions

Although the prevalent reaction mode for these intermediates is [3 + 2]-cycloadditions to imidazolidines,^8^ selective [3 + 3]-cycloadditions to the piperazine ring have been observed at the stoichiometric level in a few instances. Pioneering works in organometallic chemistry involved complexes bearing deprotonated imines ([R—CH=N—CHR′]^−^, smif-type ligands) of group 4 transition metals,^9^ Fe,^10^ Al,^11^ and Zn,^12^ which rendered binuclear complexes with a bridging dianionic piperazine. From these, free piperazines have been rarely isolated.^9b,11^ More recently, an original combination of aluminum reagents under visible-light irradiation to form piperazines has been reported.^13^ In this regard, we are not aware of previous examples of such [3 + 3]-cycloadditions at a catalytic level.

Herein, we showcase a powerful atom-economical method for the catalytic synthesis of C-substituted piperazines from formal [3 + 3]-cycloadditions of both aromatic and aliphatic imines. High yields and excellent regio- and diastereoselective control are achieved using [IrCl(cod)(PPh_3_] (**5**, cod = 1,5-cyclooctadiene) as a catalyst under mild reaction conditions.

Initial studies started analyzing the response of [{Ir(μ-Cl)(cod)}_2_] (**1**) toward Py^A^—CH=N—CH_2_Py^B^ (**2a**; Py = 2-pyridyl), which rendered the neutral complex [IrCl(cod)(Py^A^—CH=N—CH_2_Py^B^)] (**3**, Figure 2). A chelating coordination mode of the imine to iridium through the nitrogen atoms of the imine and Py^A^ is proposed in **3**, as found in related rhodium and iridium complexes.^14^ A further addition of the imine to **3** gave [Ir(cod)(κ^3^-*N*,*N*′,*N''*-HL1)]Cl ([**4**]Cl), where HL1 is an imidazolidine-type ligand (Figure 2). Most likely, the imidazolidine ring results from a 1,3-dipolar cycloaddition of the azomethine ylide moiety with the imine ([3 + 2]-cycloaddition), as described above.^8b^

**Figure 2:**
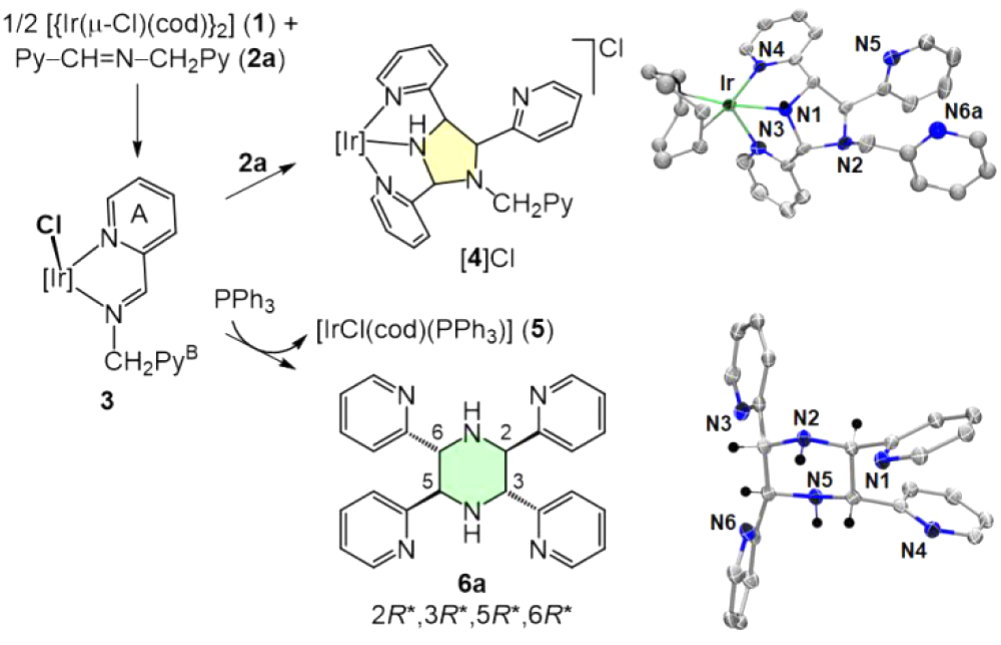
Reaction of [{Ir(μ-Cl)(cod)}_2_] (**1**) with **2a** in C_6_D_6_ to give complex **3** and subsequent reactions either with a second equivalent of **2a** to give complex [**4**]Cl or with PPh_3_ to yield [IrCl(cod)(PPh_3_)] (**5**) and **6a**. [Ir] = Ir(cod). The molecular structures (ORTEP, ellipsoids set at 50% probability) of the cation [**4**]^+^ and **6a** (2*R*,3*R*,5*R*,6*R*-enantiomer) are shown on the right. For selected bond distances and angles, see the Supporting Information.

To our delight, the addition of PPh_3_ to **3** rendered the neutral compound [IrCl(cod)(PPh_3_)] (**5**) and the piperazine **6a** (Figure 2). This reaction highlights the crucial role of PPh_3_ in providing a divergent reaction pathway that controls the regioselectivity of the reaction to the six-membered piperazine instead of to the five-membered imidazolidine.

It is also worth noting the diastereoselectivity in the synthesis of **6a**, since only one diastereoisomer was quantitatively formed, as observed by ^1^H and ^13^C{^1^H} NMR spectroscopy. Notice that the coupling of two imines renders four new C-stereocenters, so that three enantiomeric pairs and three *meso* forms could be formed *a priori* from **2a**. The absolute configuration of isolated **6a** as the 2*R*,3*R*,5*R*,6*R* and 2*S*,3*S*,5*S*,6*S* enantiomeric pair (denoted as 2R*,3R*,5R*,6R*) was determined via X-ray crystallographic analysis (Figure 2). This configuration contrasts with that in the previous known examples, which systematically rendered the related isomer 2R*,3R*,5S*,6S*.

The reaction of **3** with PPh_3_ was monitored by ^1^H NMR spectroscopy. This reaction mixture cleanly evolved to the piperazine **6a** and complex **5** over 22 h, while the uncoordinated imine **2a** along with broad resonances for the cod peaks of **5** were initially observed. Accordingly, the direct reaction between equimolar amounts of [IrCl(cod)(PPh_3_)] (**5**) and the imine **2a** yielded **6a** directly in a very good yield in 6 h (Figure S1).

At a catalytic level, using 2 mol% **5** in C_6_D_6_ at 25 °C, the reaction was found to be significantly more complicated. A mixture of imidazolidines (34%) along with only an 18% yield of the desired piperazine **6a** was obtained after 12 h of reaction (entry 1, Table 1 and Figure 3).

**Table 1:** Screening of the Reaction Conditions for the Catalytic Synthesis of **6a** from **2a**^a^

entry	solvent	additive	time (min)	conv. (%)^b^	select. (%)^b^
1	C_6_D_6_		744	52	11
2	C_6_D_6_	Na_2_CO_3_	512	90	77
3	C_6_D_6_	NEt_3_	137	94	96
4	CD_2_Cl_2_	NEt_3_	316	94	61
5	CD_3_CN	NEt_3_	187	95	72
6	C_6_D_6_	NEt_3_-dist.	242	83	80
7	C_6_D_6_	Me_3_NO·2H_2_O	93	97	94
8	CD_3_CN	Me_3_NO·2H_2_O	18	95	94
9	C_6_D_6_	C_6_H_5_NO	199	79	79
10	C_6_D_6_	TEMPO	246	68	76

a Reaction conditions: [IrCl(cod)(PPh_3_)] (**5**, 0.0084 mmol), additive (0.084 mmol), and **2a** (0.42 mmol) in solvent (total volume = 0.5 mL) at 25 °C.

b Selectivity to piperazine. Determined by ^1^H NMR spectroscopy respect to an internal standard (toluene, 0.075 mmol).

**Figure 3:**
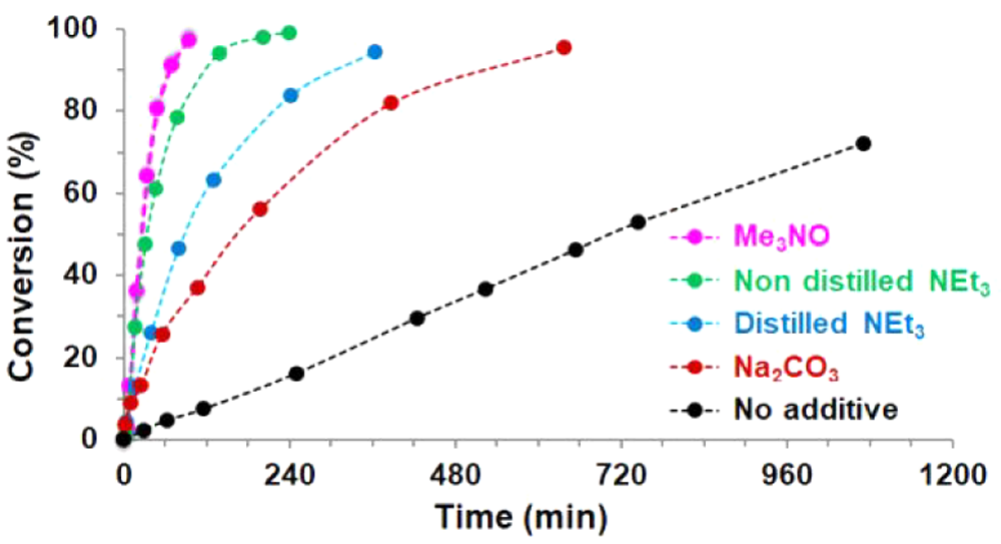
Plot of conversion (%) vs time (min) for the synthesis of piperazine **6a** catalyzed by **5** in C_6_D_6_ for Table 1 entries 1 (black), 2 (red), 3 (green), 6 (blue), and 7 (pink). Dashed lines are for visual aid.

Noticeably, the addition of a base such as Na_2_CO_3_ or NEt_3_ resulted in a significant improvement in both regioselectivity and reaction times (entries 2 and 3, Table 1). Moreover, for the more effective NEt_3_, small differences were observed in CD_3_CN, while it was found to be slower in CD_2_Cl_2_ (entries 3–5, Table 1).

Surprisingly, it was found that the addition of NEt_3_ purified by distillation resulted in a loss of the catalytic activity (entry 6, Table 1 and Figure 3). Analysis by mass spectroscopy of the unpurified NEt_3_ indicated that it contained a small amount of triethylamine *N*-oxide (<5%). Therefore, the effect of *N*-oxides was analyzed by testing the catalysis in the presence of Me_3_NO·2H_2_O, pyridine *N*-oxide (C_6_H_5_NO), and the radical *N*-oxide TEMPO (2,2,6,6-tetramethylpiperidin-1-yloxyl) (entries 7–10, respectively, Table 1).

Remarkably, the addition of 10 mol equiv Me_3_NO in C_6_D_6_ considerably reduced the reaction time (entry 7). Moreover, the use of a polar solvent, such as CD_3_CN, which increases the solubility of Me_3_NO, resulted in 95% conversion in just 18 min (entry 8, Table 1). Through this methodology, the reaction was scaled-up to a gram scale, yielding **6a** as an off-white solid with a 94% isolated yield. In the same line, C_6_H_5_NO as well as TEMPO also accelerated the reaction, albeit to a lesser extent (entries 9 and 10, respectively, Table 1).

In a parallel experiment, the reaction between [IrCl(cod)(PPh_3_)] (**5**) and Me_3_NO showed that **5** slowly converts to [IrCl(cod)(OPPh_3_)] with 18% conversion after 24 h. Therefore, it seems unlikely that this reaction has significant impact on is significantly impacted by the time scale of the catalysis. In addition, control experiments in the absence of **5** showed no conversion to piperazine with or without the presence of Me_3_NO (Table S1).

The prominent role of Me_3_NO could be derived from its expected ability to act as a hydrogen transfer reagent, as recently reported for related pyridine *N*-oxides,^15^ which would provide a low-energy pathway to the azomethine ylide intermediate (Scheme 1, 1,2-hydrogen shift). In this regard, the reduced positive effect of bases and the more active *N*-oxide radical, TEMPO, could be related to the participation of the probably less reactive anionic [Py—CH=N—CHPy]^−^ (^Py2^smif) and radical [Py—CH=N—CHPy]^●^ intermediates, respectively.

The substrate scope was investigated under the experimental conditions outlined in entry 7 (Table 1). Although for Py—CH=N—CH_2_Py the catalysis is faster in acetonitrile (entry 8, Table 1), for the rest of imines acetonitrile resulted in less selective reactions. Ultimately, the best compromise between conversion and selectivity was using C_6_D_6_ as the solvent.

As shown in Table 2, the reactions were found to be regioselective to the piperazine ring and diastereoselective to the head-to-head 2R*,3R*,5R*,6R* isomer, as confirmed by NMR spectroscopy and X-ray diffraction studies on selected piperazines (**6a**, **6d**, and **6i**, see the Supporting Information).^16^

**Table 2:**
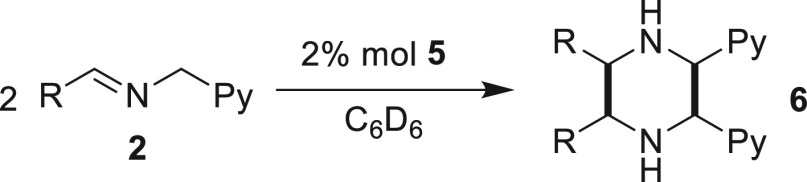

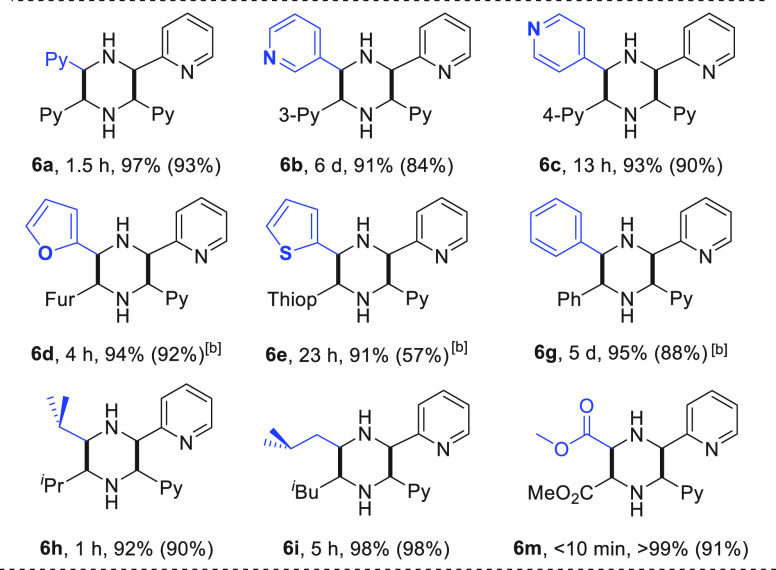
Scope of the Piperazine Synthesis.^a^

a For reaction conditions, see entry 7 in Table 1. The isolated yield is given in parentheses.

b Reactions were performed at 60 °C.

Moving the position of the N-atom on the pyridine bonded to the imine carbon using R—CH=N—CH_2_Py (R = 3-pyridyl, 4-pyridyl) allowed the preparation of piperazines **6b** and **6c**, although it was associated in increased reaction times relative to **6a**. The same applies to imines featuring the heterocycles 2-furanyl and 2-tiophenyl, which rendered **6d** and **6e** in very good yields. In the same line, Ph—CH=N—CH_2_Py (**2g**) produced **6g**, although the reaction was found to be very slow. Given the higher thermal stability of these imines (**2d**, **2e**, and **2g**), the reactions were performed at 60 °C. In the particular case of R = 2-pyrrolyl, only a trace amount of **6f** was obtained, which reveals the negative role of the acidic NH proton on the heterocycle.

Aliphatic imines, R—CH=N—CH_2_Py (R = ^*i*^Pr and ^*i*^Bu), were found to convert in a facile manner into **6h** and **6i**, respectively, with relatively short reaction times at 25 °C. Meanwhile, the reaction with the more sterically encumbered aliphatic imine, Et_2_CH–C=N–CH_2_Py (**2j**), dramatically decreased the conversion and selectivity of the reaction, yielding a mixture of expected piperazine **6j** and imidazolidines.

Unlike aromatic imines, both aliphatic imines (**2h** and **2i**) converted to the corresponding piperazines in the absence of Me_3_NO with comparable reaction times (Table S1). Such a difference could be attributed to the electron-donating nature (EDG) of the R group bonded to the imine carbon. Moreover, it was found that an activating group in the R′ position of the imine R—CH=N—CH_2_R′ is key for the success of the catalysis. Indeed, no reaction occurred with Py—CH=N—CHMe_2_ (**2k**) even after 4 days at rt. In the same line, the use of the imine Py—CH=N—CH_2_Ph (**2l**) resulted in a considerable loss in the selectivity. A mixture of the piperazine **6l** and unidentified products (ratio 1:4) was obtained after 2 days at 60 °C (57% conversion), while the imine Py—CH=N—CH_2_CO_2_Me (**2m**) resulted in a noticeable increase of the reaction rate with a 99% conversion to **6m** in less than 10 min. These results agree with an enhanced catalytic activity with imines R—CH=N—CH_2_R′ featuring electron-donating groups (EDG) bonded to the CH and electron-withdrawing groups (EWG) bonded to the CH_2_.

In conclusion, we have proved that C-substituted piperazines can be synthesized in a stereospecific and straightforward manner using an accessible iridium catalyst under mild reaction conditions. The developed method is very simple and scalable, as it only requires imines as the starting products. Furthermore, the unique diastereomer obtained has been previously unreported and indicates that a distinct reaction pathway is operating in this catalysis.

## Data Availability

Crystallographic data for piperazines **6a**, **6d**, **6i,** and for [**4**]Cl have been deposited in the Cambridge Crystallographic Data Centre (2218904–2218907).

## Supporting Information Available

Experimental details; catalytic studies; and selected NMR spectra of the complexes, imines, and piperazines (PDF)
Crystallographic data for complex [**4**]Cl (CIF)
Crystallographic data for piperazine **6a** (CIF)
Crystallographic data for piperazine **6d** (CIF)
Crystallographic data for piperazine **6i** (CIF)

## Supplementary Material

cs2c05895_si_001.pdf

cs2c05895_si_002.cif

cs2c05895_si_003.cif

cs2c05895_si_004.cif

cs2c05895_si_005.cif
