# Supplementary material for: Iridium-Catalyzed Regio- and Diastereoselective Synthesis of C-Substituted Piperazines
Source: ACS Catal. 2023 Feb 16;13(5):3148–52. doi: 10.1021/acscatal.2c05895 (PMC9990149; doi:10.1021/acscatal.2c05895)
Supplement: Supplementary file 1 — cs2c05895_si_001.pdf [file cs2c05895_si_001.pdf]

Iridium Catalyzed Regio- and Diastereo-Selective Synthesis of *C*-Substituted Piperazines

Luis Tarifa, M. Pilar del Río,[†] Laura Asensio, José A. López, Miguel A. Ciriano, Ana M. Geer,* Cristina Tejel*

Departamento de Química Inorgánica, Instituto de Síntesis Química y Catálisis Homogénea (ISQCH), CSIC-Universidad de Zaragoza, 50009 Zaragoza, Spain

e-mail: ctejel@unizar.es, anageer@unizar.es

Table of Contents

Experimental Procedures 2

Starting materials and physical methods 2

Synthesis of complexes and monitoring of reactions 2

Synthesis of imines 3

Catalytic experiments and synthesis of piperazines 5

X-ray diffraction studies on complex [Ir(cod)($C_{24}H_{22}N_6$)]Cl [4]Cl and piperazines 6a, 6d, and 6i 8

NMR spectra of complexes, imines and piperazines 10

Complexes 3 and [4]Cl 10

Imines 11

Piperazines 13

References 18

Experimental Procedures

Starting materials and physical methods

The operations were performed under an argon atmosphere using standard Schlenk techniques and glovebox facilities. Aldehydes and amines were were adquired commercially and distilled before use. C_6D_6 and CD_3CN were dried over 4 Å molecular sieves and degassed through three freeze-pump-thaw cycles. [{Ir(μ-Cl)(cod)}$_2$] (**1**) was prepared according to the literature method.[S1] All other reagents and solvents were adquired commercially and used as received unless otherwise stated. Carbon, hydrogen, and nitrogen analyses were carried out with a Perkin-Elmer 2400 CHNS/O microanalyzer. NMR spectra were recorded on Bruker AV300, AV400 and AV500 spectrometers operating at 300.13, 400.13 MHz and 500.13 MHz, respectively, for ^{1}H. Chemical shifts are reported in ppm and referenced to $SiMe_4$, using the internal signal of the deuterated solvent (^{1}H and ^{13}C). ^{31}P NMR chemical shifts are reported relative to external 85 % H_3PO_4. ^{1}H NMR spectra for quantitative measurements were recorded using the standard sequence from Bruker zg30, with 8 scans and a delay (d1) of 15 s, which ensures a good relative intensity of the selected resonances of complexes/substrates/products and that from the internal standard (Me signal of toluene). Mass spectra and high resolution mass specta of complexes were acquired on a Bruker Esquire3000 plus (ESI+) and a Bruker MicroTOF-Q (ESI+) spectrometers, respectively. Conductivities were measured in methanol solutions using a Philips PW 9501/01 conductivity meter.

Synthesis of complexes and monitoring of reactions

[IrCl(cod)(PPh$_3$)] (5): was prepared according to the modified literature method as described.[S2] [{Ir(μ-Cl)(cod)}$_2$] (**1**, 542.0 mg, 0.806 mmol) and PPh_3 (423.3 mg, 1.612 mmol) were dissolved in toluene (12 mL) and the reaction was stirred for 15 minutes yielding an orange suspension. The suspension was filtered throught celite, and the filtrate was concentrated under reduced pressure to approx. 4 mL and layered with *n*-hexane (10 mL). After 3 days, the product was isolated as orange crystals, which were separated by decantation, washed with hexane (2 x 5 mL) and vacuum-dried. Yield: 395.3 mg (82%).

[IrCl(cod)(Py–CH=N–CH$_2$Py)] (3): Py–CH=N–CH$_2$Py (**2a**, 53 μL, 0.298 mmol) was added dropwise to a solution of [{Ir(μ-Cl)(cod)}$_2$] (**1**, 100.0 mg, 0.149 mmol) in toluene (5 mL) leading to an immediate change from a red solution to a dark blue suspension. The dark blue solid was separated by filtration, washed with hexane (3 x 5 mL) and vacuum-dried. Yield: 136.5 mg (86%).

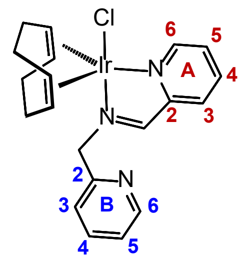

^{1}H NMR (500.13 MHz, CD_2Cl_2, –10 ºC): δ = 9.15 (s, 1H, HC=N), 8.62 (d, *J* = 4.8 Hz, 1H, H^{A6}), 8.24 (d, *J* = 5.8 Hz, 1H, H^{B6}), 7.99 (td, *J* = 7.7, 1.4 Hz, 1H, H^{B4}), 7.88 (d, *J* = 7.9 Hz, 1H, H^{B3}), 7.74 (td, *J* = 7.7, 1.9 Hz, 1H, H^{A4}), 7.53 (ddd, *J* = 7.4, 5.8, 1.6 Hz, 1H, H^{B5}), 7.47 (d, *J* = 7.8 Hz, 1H, H^{A3}), 7.29 (ddd, *J* = 7.7, 4.9, 1.1 Hz, 1H, H^{A5}), 5.05 (s, 2H, H_2C–N), 3.92 (br, 4H, =CH), 2.26 (m, 4H, CH_2^{ex}), 1.74 (m, 4H, CH_2^{en}).

^{13}C{^{1}H} NMR (125.5 MHz, CD_2Cl_2, –10 ºC): δ = 163.6 (HC=N), 157.1 (C^{A2}), 156.3 (C^{B2}), 150.0 (C^{A6}), 146.6 (C^{B6}), 137.5 (C^{A4}), 135.2 (C^{B4}), 128.0 (C^{B3}), 126.3 (C^{B5}), 124.3 (C^{A3}), 123.4 (C^{A5}), 63.1 (H_2C–N), 60.5 (=CH), 32.5 (CH_2).

HR-ESI$^+$-MS (m/z): calcd for $[IrC_{20}H_{23}N_3]^+$ $[M]^+$ 498.1516, found 498.1489 (err [mDa] = –2.7). Anal. calcd (%) for $IrC_{20}H_{23}N_3Cl$: C 45.06, H 4.35, N 7.88; found: C 45.30, H 4.34, N 7.87. Ω_M (5.0 × 10^{-4} in MeOH) = 36.0 S mol^{-1} cm^{-1}.

[Ir(cod)(C$_{24}$H$_{22}$N$_6$)]Cl [4]Cl: Py–CH=N–CH$_2$Py (**2a**, 106 μL, 0.596 mmol) was added dropwise to a solution of [{Ir(μ-Cl)(cod)}$_2$] (**1,** 100.0 mg, 0.149 mmol) in CH_2Cl_2 (10 mL) resulting in a blue suspension, which was stirred at room temperature (rt). After 24 h, the resulting yellow suspension was filtered through a celite plug, concentrated under reduced pressure to approx. 3 mL and layered with hexane (10 mL). After 3 days, needle-shaped yellow crystals were formed, which were separated by decantation, washed with hexane (2 x 5 mL) and vacuum-dried. Yield: 161.4 mg (74%).

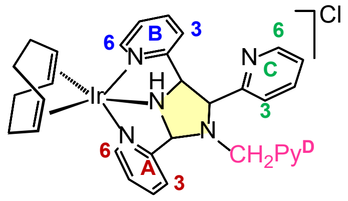

^{1}H NMR (400.13 MHz, CD_2Cl_2, 25 ºC): δ = 9.73 (dd, *J* = 9.4, 6.5 Hz, 1H, NH), 9.08 (d, *J* = 4.8 Hz, 1H, H^{B6}), 8.75 (ddd, *J* = 4.9, 1.7, 0.8 Hz, 1H, H^{C6}), 8.61 (d, *J* = 4.9 Hz, 1H, H^{A6}), 8.57 (ddd, *J* = 4.8, 1.7, 0.8 Hz, 1H, H^{D6}), 7.98 (d, *J* = 8.0 Hz, 1H, H^{A3}), 7.81 (td, *J* = 7.8, 1.5 Hz, 1H, H^{A4}), 7.78 (td, *J* = 7.7, 1.8 Hz, 1H, H^{C4}), 7.76 (d, *J* = 7.7 Hz, 1H, H^{D3}), 7.66 (td, *J* = 7.6, 1.8 Hz, 1H, H^{D4}), 7.62 (d, *J* = 7.8 Hz, 1H, H^{C3}), 7.50 (td, *J* = 7.8, 1.6 Hz, 1H, H^{B4}), 7.35 (ddd, *J* = 7.5, 4.9, 1.1 Hz, 1H, H^{C5}), 7.26 (t, *J* = 6.6 Hz, 1H, H^{A5}), 7.22 (t, *J* = 6.5 Hz, 1H, H^{B5}), 7.16 (ddd, *J* = 7.4, 4.9, 0.9 Hz, 1H, H^{D5}), 6.90 (d, *J* = 8.1 Hz, 1H, H^{B3}), 6.35 (t, *J* = 5.9 Hz, 1H, CH^B), 5.78 (d, *J* = 6.1 Hz, 1H, CH^A), 5.02 and 3.47 (AX spin ystem, *J* = 13.7 Hz, 2H, H_2C–N), 3.95 (d, *J* = 9.1 Hz, 1H, CH^C), 3.63 (td, *J* = 7.8, 2.4 Hz, 2H, =CH), 3.05 (td, *J* = 7.8, 2.6 Hz, 2H, =CH), 2.45 (m, 2H, CH_2^{ex}), 2.27 (m, 2H, CH_2^{ex}), 1.65 (m, 2H, CH_2^{en}), 1.58 (m, 2H, CH_2^{en}).

$^{13}C\{^1H\}$ NMR (101.5 MHz, CD_2Cl_2, 25 ºC): δ = 165.4 (C^{B2}), 163.3 (C^{A2}), 159.4 (C^{D2}), 154.3 (C^{C2}), 150.8 (C^{B6}), 150.2 (C^{C6}), 150.1 (C^{D6}), 148.8 (C^{A6}), 138.7 (C^{A4}), 138.1 (C^{B4}), 137.7 (C^{C4}), 137.0 (C^{D4}), 126.9 (C^{C3}), 125.9 (C^{A5}), 125.6 (C^{D3}), 125.4 (C^{A3}), 124.7 (C^{B5}), 124.3 (C^{C5}), 122.6 (C^{D5}), 122.5 (C^{B3}), 88.9 (CH^A), 75.7 (CH^C), 69.0 (CH^B), 56.8 (=CH), 56.3 (=CH), 53.6 (H_2C–N), 33.2 (CH_2), 32.9 (CH_2).

HR-ESI$^+$-MS (m/z): calcd for $[Ir(C_{32}H_{34}N_6)]^+$ $[M]^+$ 695.2470, found 695.2476 (err [mDa] = 0.6). Anal. Calcd (%) for $[Ir(C_{32}H_{34}N_6)]Cl\cdot(CH_2Cl_2)_{0.75}$: C 49.54, H 4.51, N 11.16; found: C 49.81, H 4.53, N 10.58.

Reaction of [IrCl(cod)(PPh$_3$)] (5) with Py–CH=N–CH$_2$Py (2a): 2a (2.98 µL, 0.0167 mmol) was added to an NMR tube containing complex **5** (10.0 mg, 0.0167 mmol) in C_6D_6 (0.5 mL). The reaction was monitored by ^1H NMR spectroscopy observing the quantitative conversion of the imine to the piperazine **6a** after 6 h at rt (Figure S1, b).

Reaction of [IrCl(cod)(Py–CH=N–CH$_2$Py)] (3) with PPh$_3$: PPh_3 (4.9 mg, 0.0187 mmol) was added to an NMR tube containing complex **3** (10.0 mg, 0.0187 mmol) and toluene (8 µL, 0.0752 mmol) as an internal standard in C_6D_6 (0.5 mL). The reaction was monitored by ^1H NMR spectroscopy observing the initial formation of [IrCl(cod)(PPh$_3$)] (**5**) and the imine **2a** after 14 min at rt. After 22 hours at rt, the quantitative conversion of **2a** into piperazine **6a** was observed (Figure S1, c).

Reaction of [IrCl(cod)(PPh$_3$)] (5) with Me$_3$NO•2H$_2$O: the reaction was monitored by ^1H and $^{31}P\{^1H\}$ NMR spectroscopy. After 24 h at room temperature, few amounts of [IrCl(cod)(OPPh$_3$)] (18%) were observed.

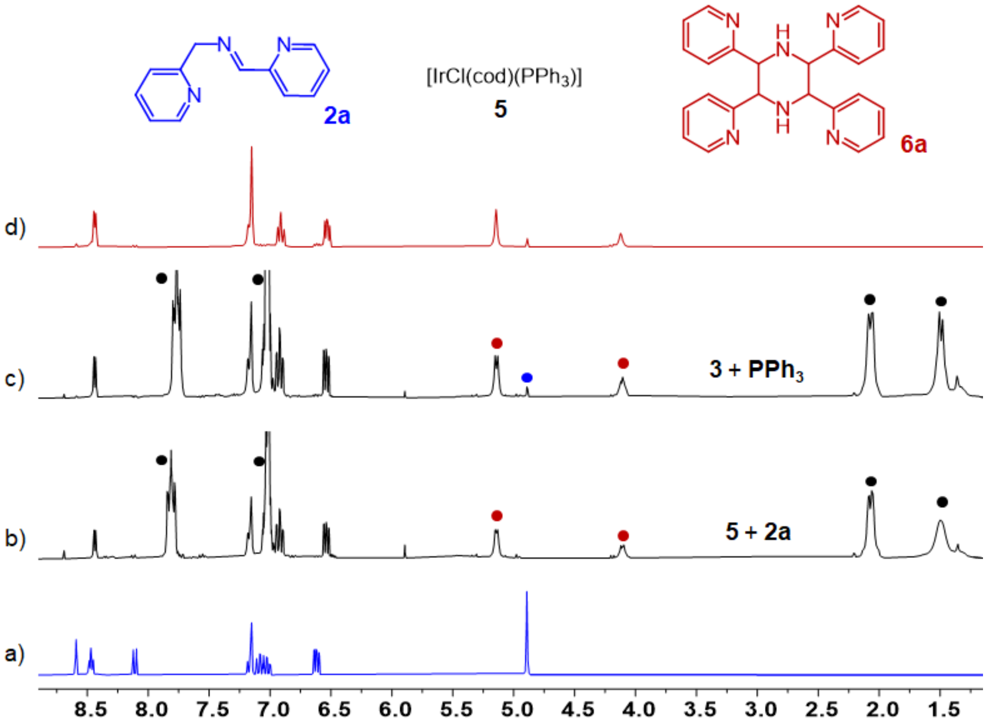

Figure S1. ^1H NMR spectra in C_6D_6 of: a) Py–CH=N–CH$_2$Py (**2a**, in blue), b) the reaction mixture of [IrCl(cod)(PPh$_3$)] (**5**) with the imine **2a** after 6 h in C_6D_6, c) the reaction mixture of [IrCl(cod)(Py–CH=N–CH$_2$Py)] (**3**) with PPh$_3$ after 22 h in C_6D_6, d) the piperazine **6a** (in red).

Synthesis of imines

General procedure for the synthesis of imines (for imines **2a**-**2c**, **2e**, **2g**-**2j**):

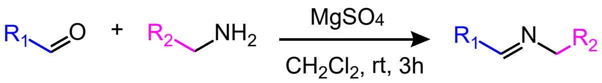

Freshly distilled equimolar amounts of the aldehyde (0.01 mol) and amine (0.01 mol) were added to a suspension of $MgSO_4$ (5.0 g, 0.0415 mol) in dichloromethane (10 mL). The reaction mixture was stirred for 3 h in the absence of light, filtered through celite and the filtrate was vacuum-dried. The imines were stored in a –20 ºC freezer until use and the ^1H NMR spectrum was checked before use. Imines **2a**,[S3] **2g**,[S4] **2e**,[S4] **2l**,[S5] and **2k**[S6] were identified by comparison to the data reported in literature.

Imines **2d**[S4] , **2f**[S7] and **2m**[S8] were prepared according to the procedure reported in the literature.

Unreported imines (or those whose NMR data is unreported, **2b**, **2c**, **2h**, **2i**, **2j**) were characterized by NMR spectroscopy and mass spectrometry and the information is detailed below. For NMR spectra of these imines see Figures S10-S19.

N-(pyridin-2-ylmethyl)-1-(pyridin-3-yl)methanimine (**2b**, isolated yield: 79%)

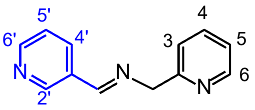

^{1}H NMR (300.13 MHz, CDCl$_3$, 25 ºC): δ = 8.92 (ddd, *J* = 2.2, 0.9 Hz, 1H, H$^{2'}$, Py'), 8.65 (dt, *J* = 4.8, 1.2 Hz, 1H, H$^{6'}$, Py'), 8.56 (ddd, *J* = 4.8, 1.9, 0.9 Hz, 1H, H^6, Py), 8.50 (t, *J* = 1.4 Hz, 1H, HC=N), 8.16 (dt, *J* = 7.9, 2.0 Hz, 1H, H$^{4'}$, Py'), 7.67 (tt, *J* = 7.6, 1.3 Hz, 1H, H^4, Py), 7.41 (dd, *J* = 7.8, 1.1 Hz, 1H, H^3, Py), 7.33 (dd, *J* = 7.9, 4.8 Hz, 1H, H$^{5'}$, Py'), 7.17 (ddd, *J* = 7.5, 4.9, 1.2 Hz, 1H, H^5, Py), 4.96 (d, *J* = 1.4 Hz, 2H, H$_2$C–N).

^{13}C{^{1}H} NMR (75.5 MHz, CDCl$_3$, 25 ºC): δ = 160.2 (HC=N), 158.7 (C^2, Py), 151.7 (C$^{6'}$, Py'), 150.4 (C$^{2'}$, Py'), 149.4 (C^6, Py), 136.8 (C^4, Py), 134.7 (C$^{4'}$, Py'), 131.6 (C$^{3'}$, Py'), 123.7 (C$^{5'}$, Py'), 122.4 (C^3, Py), 122.2 (C^5, Py), 67.0 (H$_2$C–N).

HR-ESI$^+$-MS (m/z): calcd. for $C_{12}H_{11}N_3Na$ [*M* + *Na*]$^+$ 222.0845, found 222.0844 (err [mDa] = –0.1).

N-(pyridin-2-ylmethyl)-1-(pyridin-4-yl)methanimine (**2c**, isolated yield: 78%)

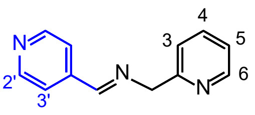

^{1}H NMR (300.13 MHz, CDCl$_3$, 25 ºC): δ = 8.70 (d, *J* = 6.0 Hz, 1H, H$^{2'}$, Py'), 8.58 (dd, *J* = 4.8, 1.9 Hz, 1H, H^6, Py), 8.46 (d, *J* = 1.5 Hz, 1H, HC=N), 7.69 (td, *J* = 7.7, 1.8 Hz, 1H, H^4, Py), 7.65 (d, *J* = 6.0 Hz, 1H, H$^{3'}$, Py'), 7.42 (d, *J* = 7.8 Hz, 1H, H^3, Py), 7.20 (ddd, *J* = 7.5, 4.9, 1.2 Hz, 1H, H^5, Py), 4.99 (d, *J* = 1.5 Hz, 2H, H$_2$C–N).

^{13}C{^{1}H} NMR (75.5 MHz, CDCl$_3$, 25 ºC): δ = 160.7 (HC=N), 158.0 (C^2, Py), 150.1 (C$^{2'}$, Py'), 149.1 (C^6, Py), 142.4 (C$^{4'}$, Py'), 136.4 (C^4, Py), 122.1 (C^3, Py), 121.9 (C^5, Py), 121.7 (C$^{3'}$, Py'), 66.5 (H$_2$C–N).

HR-ESI$^+$-MS (m/z): calcd. for $C_{12}H_{11}N_3Na$ [*M* + *Na*]$^+$ 222.0845, found 222.0835 (err [mDa] = –1.0).

2-methyl-N-(pyridin-2-ylmethyl)propan-1-imine (**2h**, isolated yield: 77%)

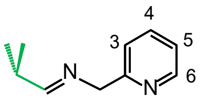

^{1}H NMR (300.13 MHz, CDCl$_3$, 25 ºC): δ = 8.51 (ddd, *J* = 4.9, 1.9, 0.9 Hz, 1H, H^6, Py), 7.72 (dt, *J* = 4.9, 1.4 Hz, 1H, HC=N), 7.62 (td, *J* = 7.7, 1.9 Hz, 1H, H^4, Py), 7.30 (d, *J* = 7.8, 1H, H^3, Py), 7.12 (ddd, *J* = 7.6, 4.9, 0.7 Hz, 1H, H^5, Py), 4.67 (s, 2H, H$_2$C–N), 2.50 (sept, *J* = 6.8 Hz, 1H, CH, ^{i}Pr), 1.1 (d, *J* = 6.9 Hz, 6H, Me, ^{i}Pr).

^{13}C{^{1}H} NMR (75.5 MHz, CDCl$_3$, 25 ºC): δ = 172.3 (HC=N), 159.5 (C^2, Py), 149.3 (C^6, Py), 136.6 (C^4, Py), 122.0 (C^3, Py), 121.9 (C^5, Py), 66.6 (H$_2$C–N), 34.3 (CH, ^{i}Pr), 19.4 (Me, ^{i}Pr).

HR-ESI$^+$-MS (m/z): calcd. for $C_{10}H_{15}N_2$ [*M* + *H*]$^+$ 163.1230, found 163.1234 (err [mDa] = 0.4).

3-methyl-N-(pyridin-2-ylmethyl)butan-1-imine (**2i**, isolated yield: 79%)

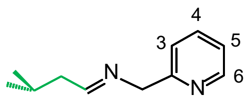

^{1}H NMR (300.13 MHz, CDCl$_3$, 25 ºC): δ = 8.47 (ddd, *J* = 4.9, 1.9, 1.0 Hz, 1H, H^6, Py), 7.80 (tt, *J* = 5.2, 1.4 Hz, 1H, HC=N), 7.57 (td, *J* = 7.7, 1.9 Hz, 1H, H^4, Py), 7.26 (d, *J* = 7.8 Hz, 1H, H^3, Py), 7.07 (dddd, *J* = 7.7, 4.8, 1.3, 0.7 Hz, 1H, H^5, Py), 4.64 (s, 2H, H$_2$C–N), 2.16 (dd, *J* = 7.1, 5.2 Hz, 2H, CH$_2$, ^{i}Bu), 1.91 (sept, *J* = 6.7 Hz, 1H, CH, ^{i}Bu), 0.90 (d, *J* = 6.7 Hz, 6H, Me, ^{i}Bu).

^{13}C{^{1}H} NMR (75.5 MHz, CDCl$_3$, 25 ºC): δ = 167.2 (HC=N), 159.2 (C^2, Py), 149.2 (C^6, Py), 136.6 (C^4, Py), 122.1 (C^3, Py), 121.9 (C^5, Py), 66.9 (H$_2$C–N), 44.9 (CH$_2$, ^{i}Bu), 26.3 (CH, ^{i}Bu), 22.5 (Me, ^{i}Bu).

HR-ESI$^+$-MS (m/z): calcd. for $C_{11}H_{17}N_2$ [*M* + *H*]$^+$ 177.1386, found 177.1389 (err [mDa] = 0.3).

2-ethyl-N-(pyridin-2-ylmethyl)butan-1-imine (**2j**, isolated yield: 81%)

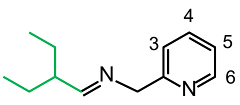

^{1}H NMR (300.13 MHz, CDCl$_3$, 25 ºC): δ = 8.49 (dd, *J* = 4.9, 1.1 Hz, 1H, H^6, Py), 7.64 – 7.54 (m, 1H, H^4, Py and 1H, HC=N), 7.29 (d, *J* = 7.8 Hz, 1H, H^3, Py), 7.09 (ddtt, *J* = 7.4, 4.9, 1.2, 0.6 Hz, 1H, H^5, Py), 4.68 (d, *J* = 1.5 Hz, 2H, H$_2$C–N), 2.09 (dt, *J* = 12.5, 6.4 Hz, 1H, CH), 1.47 (pd, *J* = 7.1, 1.3 Hz, 4H, CH$_2$, Et), 0.86 (td, *J* = 7.4, 1.4 Hz, 6H, Me , Et).

^{13}C{^{1}H} NMR (75.5 MHz, CDCl$_3$, 25 ºC): δ = 171.5 (HC=N), 159.5 (C^2, Py), 149.2 (C^6, Py), 136.6 (C^4, Py), 122.0 (C^3, Py), 121.9 (C^5, Py), 66.9 (H$_2$C–N), 48.5 (CH), 24.9 (CH$_2$, Et), 11.7 (Me, Et).

HR-ESI$^+$-MS (m/z): calcd. for $C_{12}H_{19}N_2$ [*M* + *H*]$^+$ 191.1543, found 191.1549 (err [mDa] = 0.6).

Catalytic experiments and synthesis of piperazines

Standard procedure for the synthesis of 6a with an additive. In a glovebox, complex **5** (5.0 mg, 0.0084 mmol) and the corresponding additive (0.084 mmol) were weighed into an NMR tube and dissolved in the appropriate amount of C_6D_6 to get a final volume of 0.5 mL. Then, the internal standard (toluene, 8.0 μL, 0.075mmol) and **2a** (74 μL, 0.418 mmol) were added. The NMR tube was sealed and shaken, and at this point the chronometer was switched on. The tube was taken out of the glovebox and immediately loaded into the NMR spectrometer (around 3-5 min). ^{1}H NMR spectra of the mixture were recorded at different intervals of time. The time at which each spectrum was acquired was directly taken from the Mestre-software (view/table/parameters). See Table 1 and Figure 3 in the main text for details.

Following a similar procedure, the influence of the solvent was analyzed in selected cases, and the results are depicted in Figure S2. Using $Me_3NO{\bullet}2H_2O$ as the additive, a faster reaction in acetonitrile than in benzene was observed, probably due to a better solubility of the amine oxide in acetonitrile. However, in the presence of NEt_3, a non-polar solvent such as benzene produced the best results.

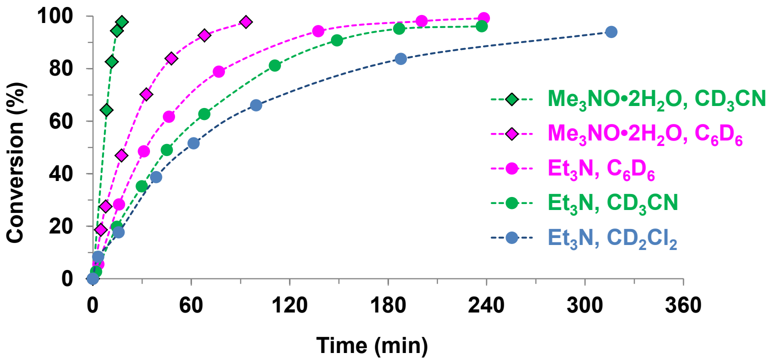

Figure S2. Conversion (%) vs. time (min) for the synthesis of piperazine **6a** with 2 mol% cat. **5** in C_6D_6 (pink), CD_3CN (green) and CD_2Cl_2 (blue) in the presence of NEt_3 (circles) and $Me_3NO{\bullet}2H_2O$ (diamonds) at 25 °C. Dashed lines are for visual aid.

Standard procedure for the synthesis of tetrasubstituted piperazines (6a-6i) with $Me_3NO{\bullet}2H_2O$. In a glovebox, complex **5** (5.0 mg, 0.0084 mmol) and $Me_3NO{\bullet}2H_2O$ (9.3 mg, 0.084 mmol) were weighed into an NMR tube and dissolved in the appropriate amount of C_6D_6 to get a final volume of 0.5 mL. Then, the internal standard (toluene, 8.0 μL), and the imine (0.418 mmol) were added. The NMR tube was sealed and shaken. At this point, the chronometer was switched on, the tube was taken out of the glovebox and immediately loaded into the NMR spectrometer (around 3-5 min). ^{1}H NMR spectra were recorded at different intervals of time; the time at which each spectrum was acquired was directly taken from the Mestre-software (view/table/parameters). All reactions were monitored by NMR spectroscopy (Figure S3). After that, the solutions were filtered through an alumina plug and washed with chloroform (3 mL). The filtrate was vacuum-dried to yield the piperazines, isolated generally as oils. For NMR spectra of the piperazines see Figures S20-S37.

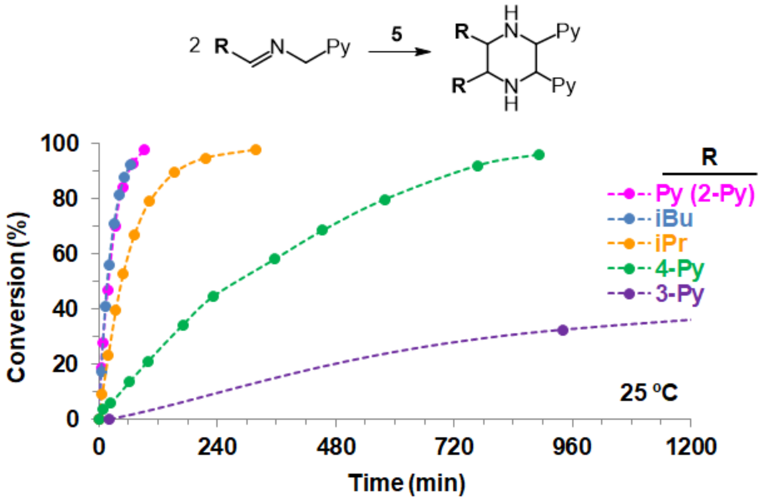

Figure S3. Conversion (%) *vs* time (min) for the synthesis of piperazines **6a** (pink), **6b** (purple), **6c** (green), **6h** (orange) and **6i** (blue) at 25 ºC. Dashed lines are for visual aid.

In the absence of catalyst, no conversion to the corresponding piperazines was observed either in the presence or absence of Me_3NO (entries 1-4, Table S1). In the particular case of imines **2h** and **2i**, they converted to the corresponding piperazines in the absence of Me_3NO with comparable reaction times (entries 5-8, Table S1).

Table S1. Additional experiments.[a]

Entry	Cat. (mol%)	Imine	Additive	Piperazine	Time (h)	Conv. (%)[b]	Select. (%)
1	---	**2a**	---	**6a**	21.0	14	0
2	---	**2a**	$Me_3NO•2H_2O$	**6a**	17.2	11	0
3	---	**2i**	-	**6i**	24.0	4	0
4	---	**2i**	$Me_3NO•2H_2O$	**6i**	24.0	4	0
5	**5** (2)	**2h**	---	**6h**	6	97	>95
6	**5** (2)	**2h**	$Me_3NO•2H_2O$	**6h**	5	98	98
7	**5** (2)	**2i**	---	**6i**	0.8	94	>95
8	**5** (2)	**2i**	$Me_3NO•2H_2O$	**6i**	1	92	90

[a] Reaction conditions: **5** (0.0084 mmol), additive (0.084 mmol), and imine (0.42 mmol) in C_6D_6 (total volume = 0.5 mL) at 25 ºC. [b] Determined by ^{1}H NMR spectroscopy respect to internal standard (toluene, 0.075 mmol).

Scaled-up procedure for the synthesis of 6a. Complex **5** (50.0 mg, 0.084 mmol) and $Me_3NO•2H_2O$ (93.0 mg, 0.84 mmol) were weighed into a Schlenk flask and dissolved in CH_3CN (5 ml). **2a** (745 µL, 4.18 mmol) was then added, the resulting suspension was stirred for 30 min and then vacuum-dried yielding a yellow oil. The product was extracted with chloroform, and the extract was filtered through alumina and vacuum-dried. Addition of diethyl ether (4 mL) to the oily residue and drying under vacuum afforded **6a** as a beige solid. Yield: 772.8 mg (93%)

***R**,*R**,*R**,*R**-2,3,5,6-tetra(pyridin-2-yl)piperazine** (**6a**, isolated yield: 93%)

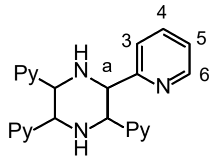

^{1}H NMR (300.13 MHz, $CDCl_3$, 25 ºC): δ = 8.56 (ddd, *J* = 4.9, 1.9, 0.9 Hz, 4H, H^6, Py), 7.49 (td, *J* = 7.7, 1.9 Hz, 4H, H^4, Py), 7.21 (d, *J* = 7.8 Hz, 4H, H^3, Py), 7.09 (ddd, *J* = 7.5, 4.9, 1.2 Hz, 4H, H^5, Py), 4.71 (s, 4H, CH^a), 3.52 (br s, 2H, NH).

^{13}C{^{1}H} NMR (75.5 MHz, $CDCl_3$, 25 ºC): δ = 161.4 (C^2, Py), 149.1 (C^6, Py), 136.1(C^4, Py), 123.3 (C^3, Py), 121.9 (C^5, Py), 58.4 (CH^a).

HR-ESI$^+$-MS (m/z): calcd. for $C_{24}H_{23}N_6$ $[M + H]^+$ 395.1978, found 395.1965 (err [mDa] = –1.3).

***R**,*R**,*R**,*R**-2,3-di(pyridin-2-yl)-5,6-di(pyridin-3-yl)piperazine** (**6b**, isolated yield: 84%)

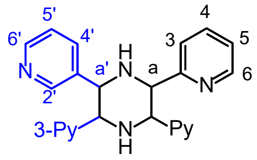

^{1}H NMR (300.13 MHz, $CDCl_3$, 25 ºC): δ = 8.65 (ddd, *J* = 4.8, 1.8, 0.9 Hz, 2H, H^6, Py), 8.33 (dd, *J* = 4.8, 1.7 Hz, 2H, H$^{6'}$, Py'), 8.17 (dd, *J* = 2.3, 0.9 Hz, 2H, H$^{2'}$, Py'), 7.89 (dt, *J* = 8.0, 1.1 Hz, 2H, H^3, Py), 7.77 (td, *J* = 7.7, 1.8 Hz, 2H, H^4, Py), 7.32 (dt, *J* = 7.9, 2.0 Hz, 2H, H$^{4'}$, Py'), 7.24 (ddd, *J* = 7.4, 2.6, 1.1 Hz, 2H, H^5, Py), 7.02 (ddd, *J* = 7.8, 4.8, 0.9 Hz, 2H, H$^{5'}$, Py'), 4.95 (s, 2H, CH^a), 3.92 (s, 2H, CH$^{a'}$), 3.02 (br s, 2H, NH).

^{13}C{^{1}H} NMR (75.5 MHz, $CDCl_3$, 25 ºC): δ = 161.8 (C^2, Py), 149.7 (C$^{2'}$, Py'), 149.3 (C^6, Py), 149.0 (C$^{6'}$, Py'), 136.7 (C$^{3'}$, Py'), 136.6 (C^4, Py), 135.5 (C$^{4'}$, Py'), 123.1 (C$^{5'}$, Py'), 122.6 (C^3, Py), 122.1 (C^5, Py), 59.5 (CH$^{a'}$), 57.4 (CH^a).

HR-ESI$^+$-MS (m/z): calcd. for $C_{24}H_{23}N_6$ $[M + H]^+$ 395.1978, found 395.1971 (err [mDa] = –0.7).

R^*,R^*,R^*,R^*-2,3-di(pyridin-2-yl)-5,6-di(pyridin-4-yl)piperazine (**6c**, isolated yield: 90%)

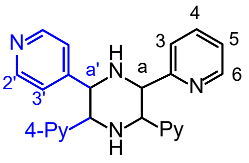

^{1}H NMR (300.13 MHz, $CDCl_3$, 25 ºC): δ = 8.65 (dt, *J* = 4.8, 1.4 Hz, 2H, H^6, Py), 8.36 (m, 4H, $H^{2'}$, Py'), 7.77 – 7.72 (m, 2H, H^3, Py and 2H, H^4, Py), 7.24 (dd, *J* = 8.9, 4.4 Hz, 2H, H^5, Py), 6.93 (m, 4H, $H^{3'}$, Py'), 4.89 (s, 2H, CH^a), 3.91 (s, 2H, $CH^{a'}$), 2.97 (br s, 2H, NH).

^{13}C{^{1}H} NMR (75.5 MHz, $CDCl_3$, 25 ºC): δ = 161.5 (C^2, Py), 149.9 ($C^{4'}$, Py'), 149.8 ($C^{2'}$, Py'), 149.3 (C^6, Py), 136.7 (C^4, Py), 123.1 ($C^{3'}$, Py'), 122.7(C^3, Py), 122.3 (C^5, Py), 60.3 ($CH^{a'}$), 57.3 (CH^a).

HR-ESI$^+$-MS (m/z): calcd. for $C_{24}H_{23}N_6$ [*M* + *H*]$^+$ 395.1978, found 395.1978 (err [mDa] = 0.0).

R^*,R^*,R^*,R^*-2,3-di(furan-2-yl)-5,6-di(pyridin-2-yl)piperazine (**6d**, isolated yield: 92%)

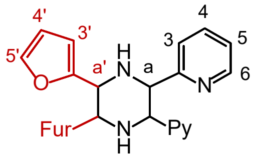

^{1}H NMR (300.13 MHz, $CDCl_3$, 25 ºC): δ = 8.48 (ddd, *J* = 4.9, 1.9, 0.9 Hz, 2H, H^6, Py), 7.47 – 7.38 (m, 4H, H^4, Py and $H^{5'}$, Fur), 7.06 (ddd, *J* = 7.5, 4.9, 1.2 Hz, 2H, H^5, Py), 6.92 (dt, *J* = 7.8, 1.2 Hz, 2H, H^3, Py), 6.48 (d, *J* = 3.2 Hz, 2H, $H^{3'}$, Fur), 6.40 (dd, *J* = 3.2, 1.9 Hz, 2H, $H^{4'}$, Fur), 4.64 (s, 2H, $CH^{a'}$), 4.40 (s, 2H, CH^a), 3.02 (br s, 2H, NH).

^{13}C{^{1}H} NMR (75.5 MHz, $CDCl_3$, 25 ºC): δ = 160.2 (C^2, Py), 155.2 ($C^{2'}$, Fur), 149.2 (C^6, Py), 141.7 ($C^{5'}$, Fur), 136.0 (C^4, Py), 123.4 (C^3, Py), 122.3 (C^5, Py), 110.5 ($C^{4'}$, Fur), 107.4 ($C^{3'}$, Fur), 60.3 ($CH^{a'}$), 52.0 (CH^a).

HR-ESI$^+$-MS (m/z): calcd. for $C_{22}H_{21}N_4O_2$ [*M* + *H*]$^+$ 373.1659, found 373.1658 (err [mDa] = –0.1).

R^*,R^*,R^*,R^*-2,3-di(pyridin-2-yl)-5,6-di(thiophen-2-yl)piperazine (**6e**, isolated yield: 57%)

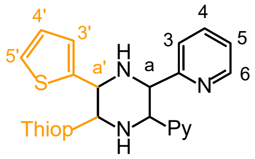

^{1}H NMR (300 MHz, $CDCl_3$, 25 ºC): δ = 8.51 (ddd, *J* = 4.8, 1.8, 0.9 Hz, 2H, H^6, Py), 7.54 (td, *J* = 7.7, 1.8 Hz, 2H, H^4, Py), 7.31 (d, *J* = 7.9 Hz, 2H, H^3, Py), 7.23 (dd, *J* = 5.1, 1.2 Hz, 2H, $H^{5'}$, Thiop), 7.11 (ddd, *J* = 7.5, 4.9, 1.2 Hz, 2H, H^5, Py), 6.95 (dd, *J* = 5.1, 3.5 Hz, 2H, $H^{4'}$, Thiop), 6.91 (dd, *J* = 3.1 Hz, 2H, $H^{3'}$, Thiop), 4.64 (s, 2H, CH^a), 4.61 (s, 2H, $CH^{a'}$), 3.07 (br s, 2H, NH).

^{13}C{^{1}H} NMR (126 MHz, $CDCl_3$, 25 ºC): δ = 160.7 (C^2, Py), 149.2 (C^6, Py), 146.3 ($C^{2'}$, Thiop), 136.2 (C^4, Py), 126.7 ($C^{4'}$, Thiop), 124.9 ($C^{3'}$, Thiop), 124.8 ($C^{5'}$, Thiop), 123.2 (C^3, Py), 122.2 (C^5, Py), 59.3 (CH^a), 56.4 ($CH^{a'}$).

HR-ESI$^+$-MS (m/z): calcd. for $C_{22}H_{21}N_4S_2$ [*M* + *H*]$^+$ 405.1202, found 405.1200 (err [mDa] = – 0.2).

R^*,R^*,R^*,R^*-2,3-diphenyl-5,6-di(pyridin-2-yl)piperazine (**6g**, isolated yield: 88%)

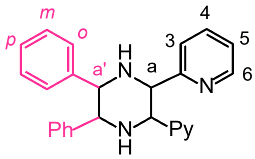

^{1}H NMR (300.13 MHz, $CDCl_3$, 25 ºC): δ = 8.67 (ddd, *J* = 4.8, 1.8, 0.9 Hz, 2H, H^6, Py), 7.97 (d, *J* = 7.8 Hz, 2H, H^3, Py), 7.75 (td, *J* = 7.7, 1.8 Hz, 2H, H^4, Py), 7.24 (ddd, *J* = 7.5, 2.6, 1.1 Hz, 2H, H^5, Py), 7.08 (m, 4H, H^m, Ph and 2H, H^p,Ph), 6.98 (m, 4H, H^o, Ph), 5.00 (s, 2H, CH^a), 3.90 (s, 2H, $CH^{a'}$), 2.95 (br s, 2H, NH).

^{13}C{^{1}H} NMR (75.5 MHz, $CDCl_3$, 25 ºC): δ = 162.7 (C^2, Py), 149.2 (C^6, Py), 142.1(C^i, Ph), 136.5 (C^4, Py), 128.1 (C^m, Ph), 127.9 (C^p, Ph), 127.2 (C^o, Ph), 122.8 (C^3, Py), 121.9 (C^5, Py), 61.6 ($CH^{a'}$). 57.7 (CH^a).

HR-ESI$^+$-MS (m/z): calcd. for $C_{26}H_{25}N_4$ [*M* + *H*]$^+$ 393.2074, found 393.2092 (err [mDa] = 1.8).

R^*,R^*,R^*,R^*-2,3-diisopropyl-5,6-di(pyridin-2-yl)piperazine (**6h**, isolated yield: 95%)

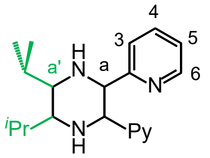

^{1}H NMR (300.13 MHz, $CDCl_3$, 25 ºC): δ = 8.49 (ddd, *J* = 4.9, 1.8, 0.9 Hz, 2H, H^6, Py), 7.38 (td, *J* = 7.6, 1.8 Hz, 2H, H^4, Py), 7.03 (ddd, *J* = 7.6, 4.9, 1.2 Hz, 2H, H^5, Py), 6.83 (dt, *J* = 7.8, 1.1 Hz, 2H, H^3, Py), 4.14 (s, 2H, CH^a), 2.63 – 2.49 (m, 2H, CH, ^{i}Pr), 2.48 (s, 2H, $CH^{a'}$), 2.30 (br s, 2H, NH), 1.06 (d, *J* = 6.4 Hz, 6H, Me, ^{i}Pr), 0.99 (d, *J* = 6.3 Hz, 6H, Me, ^{i}Pr).

^{13}C{^{1}H} NMR (75.5 MHz, $CDCl_3$, 25 ºC): δ = 160.8 (C^2, Py), 149.3 (C^6, Py), 136.0 (C^4, Py), 123.3 (C^3, Py), 122.0 (C^5, Py), 60.4 (CH^a), 58.6 ($CH^{a'}$), 26.4 (CH, ^{i}Pr), 21.1 (Me, ^{i}Pr), 20.1 (Me, ^{i}Pr).

HR-ESI$^+$-MS (m/z): calcd. for $C_{20}H_{29}N_4$ [*M* + *H*]$^+$ 325.2387, found 325.2391 (err [mDa] = 0.4).

R*,R*,R*,R*-2,3-diisobutyl-5,6-di(pyridin-2-yl)piperazine** (**6i**, isolated yield: 90%)

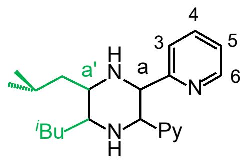

^{1}H NMR (300.13 MHz, $CDCl_3$, 25 °C): δ = 8.50 (ddd, *J* = 4.9, 1.8, 0.9 Hz, 2H, H^6, Py), 7.37 (td, *J* = 7.7, 1.8 Hz, 2H, H^4, Py), 7.03 (ddd, *J* = 7.5, 4.9, 1.2 Hz, 2H, H^5, Py), 6.84 (dt, *J* = 7.8, 1.1 Hz, 2H, H^3, Py), 4.25 (s, 2H, CH^a), 2.70 (t, *J* = 6.2, 2H, $CH^{a'}$), 2.44 (br s, 2H, NH), 1.91 – 1.64 (m, 2H, CH, and 2H, CH_2, ^{i}Bu), 1.53 (m, 2H, CH_2, ^{i}Bu), 0.95 (d, *J* = 6.4 Hz, 6H, Me, ^{i}Bu), 0.90 (d, *J* = 6.4 Hz, 6H, Me, ^{i}Bu).

$^{13}C\{^1H\}$ NMR (75.5 MHz, $CDCl_3$, 25 °C): δ = 160.9 (C^2, Py), 149.2 (C^6, Py), 135.9 (C^4, Py), 123.3 (C^3, Py), 122.0 (C^5, Py), 59.6 (CH^a), 52.3 ($CH^{a'}$), 41.4 (CH_2, ^{i}Bu), 25.1 (CH, ^{i}Bu), 23.4 (Me, ^{i}Bu), 22.6 (Me, ^{i}Bu).

HR-ESI$^+$-MS (m/z): calcd. for $C_{22}H_{33}N_4$ $[M + H]^+$ 353.2700, found 353.2703 (err [mDa] = 0.3).

R*,R*,R*,R- dimethyl 5,6-di(pyridin-2-yl)piperazine-2,3-dicarboxylate** (**6m**)

isolated yield: 91% (**6m** + **6m'**)

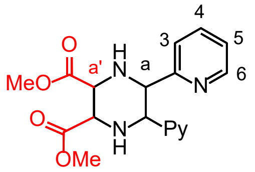

6m: ^{1}H NMR (500 MHz, C_6D_6, 25 °C): δ = 8.28 (tdd, *J* = 4.8, 1.9, 1.0 Hz, 2H, H^6, Py), 7.19 – 7.15 (m, 2H, H^3, Py), 6.98 (td, *J* = 7.6, 1.8 Hz, 2H, H^5, Py), 6.56 (ddd, *J* = 7.5, 4.8, 1.2 Hz, 2H, H^4, Py), 4.83 (s, 2H, CH^a), 4.28 (d, *J* = 3.5, 2H, $CH^{a'}$), 3.39 (s, 6H, OMe).

$^{13}C\{^1H\}$ NMR (126 MHz, C_6D_6, 25 °C): δ = 172.6 (CO), 160.7 (C^2, Py), 148.9 (C^6, Py), 135.9 (C^5, Py), 123.3 (C^3, Py), 122.0 (C^4, Py), 61.3 (CH^a), 57.3 ($CH^{a'}$), 51.8 (Me).

6m': ^{1}H NMR (500 MHz, C_6D_6, 25 °C): δ = 8.31 – 8.24 (m, 2H, H^6, Py), 7.19 – 7.15 (m, 2H, H^3, Py), 6.92 (td, *J* = 7.7, 1.9 Hz, 2H, H^5, Py), 6.50 (ddd, *J* = 7.5, 4.8, 1.2 Hz, 2H, H^4, Py), 5.00 (s, 2H, CH^a), 4.37 (d, *J* = 3.5, 2H, $CH^{a'}$), 3.37 (s, 6H, OMe).

Selected signals $^{13}C\{^1H\}$ NMR (126 MHz, C_6D_6, 25 °C): 58.8 (CH^a), 56.8 ($CH^{a'}$).

X-ray diffraction studies on complex [Ir(cod)($C_{24}H_{22}N_6$)]Cl [4]Cl and piperazines 6a, 6d, and 6i

Intensity measurements were collected with a Siemens Smart Apex (**6a** and **6i**) or a Bruker D8 Venture ([**4**]Cl and **6d**) diffractometers, with MoK_α radiation at 100 K. A semi-empirical absorption correction was applied to the data sets with the multi-scan[S9] methods. The structures were solved by direct methods with SHELX-97 (**6a** and **6i**) or SHELXT-2014[S10] ([**4**]Cl and **6d**) and refined by full-matrix least-squares on F^2 with the program SHELXL-2016,[S11] in the WINGX[S12] package. All non-hydrogen atoms were refined with anisotropic displacement parameters, except in the disordered part of [**4**]Cl. In the models of **6a** and **6d**, the hydrogen atoms were located in difference-Fourier maps and refined free, including the isotropic displacement parameters. In **6i** the hydrogen atoms were geometrically calculated and refined by the riding mode including the isotropic displacement parameters, except the hydrogens bonded to nitrogen atoms that were located in a difference-Fourier map and refined by the riding mode, but with free isotropic displacement parameters. In compound [**4**]Cl, the procedure SQUEEZE[S13] was used to model a severe solvent disorder, accounting for one hexane molecule per unit cell. Besides that, several atoms in the main molecule also are disordered; these disordered atoms were refined with isotropic displacement parameters and geometrical constraints, and the corresponding hydrogen atoms were not included in the model. The remaining hydrogen atoms were geometrically calculated and refined by the riding mode including the isotropic displacement parameters. Crystallographic data for complex [Ir(cod)($C_{24}H_{22}N_6$)]Cl ([**4**]Cl, CCDC 2218906) and piperazines **6a** (CCDC 2218904), **6d** (CCDC 2218905), and **6i** (CCDC 2218907) have been submitted to the CCDC.

Selected Crystallographic Data for [Ir(cod)($C_{24}H_{22}N_6$)]Cl ([**4**]Cl) (*local name*: jal2vs_sq): Crystal data for [4]Cl·½C_6H_{14}: $C_{32}H_{34}ClIrN_6$· ½C_6H_{14}, *Mr* = 772.38, triclinic, space group P-1, *a* = 10.8023(6), *b* = 12.6352(7), *c* = 12.6472(7) Å, α = 99.557(2), β = 105.470(2), γ = 104.374(2)°, *V* = 1560.92(15) Å^3, *Z* = 2, ρ_{calcd} = 1.643 g cm^{-3}, *F*(000) = 772, *T* = 100(2) K, MoK_α radiation (λ = 0.71073 Å, μ = 4.398 mm^{-1}). Data were collected with a yellow irregular block (0.080 × 0.040 × 0.015 mm). Of 144445 measured reflections (2θ: 4.1-54.0°), 6815 were unique (R_{int} = 0.0986). Final agreement factors were *R*1 = 0.0326 (5853 observed reflections) and *wR*2 = 0.0873. Data/restrains/parameters 6815/13/311; GOF = 1.139. Largest peak and hole in the final difference map 1.614 and -1.652 e Å^{-3}.

Selected Crystallographic Data for **6a** (*local name*: ctb91as): Crystal data: $C_{24}H_{22}N_6$, *Mr* = 394.47, monoclinic, space group P2$_1$/n, *a* = 14.1519(17), *b* = 8.8792(11), *c* = 15.8784(19) Å, β = 101.411(2), *V* = 1955.8(4) Å^3, *Z* = 4, ρ_{calcd} = 1.340 g cm^{-3}, *F*(000) = 832, *T* = 100(2) K, MoK_α radiation (λ = 0.71073 Å, μ = 0.083 mm^{-1}). Data were collected with a colourless prismatic block (0.37 × 0.34 × 0.11 mm). Of 12659 measured reflections (2θ: 4.3-54.0°), 4200 were unique

(R_{int} = 0.0251). Final agreement factors were $R1$ = 0.0391 (3453 observed reflections) and $wR2$ = 0.1075. Data/restrains/parameters 4200/0/359; GOF = 1.026. Largest peak and hole in the final difference map 0.317 and -0.241 e Å^{-3}.

Selected Crystallographic Data for **6d** (*local name*: jal8vs): Crystal data: $C_{22}H_{20}N_4O_2$, *M*r = 374.42, orthorhombic, space group P2$_1$2$_1$2, *a* = 13.9644(4), *b* = 7.0646(2), *c* = 9.3213(3) Å, *V* = 919.57(5) Å^3, *Z* = 2, ρ_{calcd} = 1.345 g cm^{-3}, *F*(000) = 392, *T* = 100(2) K, MoK$_\alpha$ radiation (λ = 0.71073 Å, μ = 0.089 mm^{-1}). Data were collected with a yellow irregular block (0.18 × 0.18 × 0.06 mm). Of 15376 measured reflections (2θ: 4.4-56.6º), 2290 were unique (R_{int} = 0.0384). Final agreement factors were $R1$ = 0.0292 (2230 observed reflections) and $wR2$ = 0.0780. Data/restrains/parameters 2290/0/167; GOF = 1.058. Largest peak and hole in the final difference map 0.252 and -0.163 e Å^{-3}.

Selected Crystallographic Data for **6i** (*local name*: ctb114as): Crystal data: $C_{22}H_{32}N_4$, *M*r = 352.51, monoclinic, space group P2$_1$/c, *a* = 12.2887(9), *b* = 17.1325(12), *c* = 10.5798(7) Å, β = 113.0110(10), *V* = 2050.2(2) Å^3, *Z* = 4, ρ_{calcd} = 1.142 g cm^{-3}, *F*(000) = 768, *T* = 100(2) K, MoK$_\alpha$ radiation (λ = 0.71073 Å, μ = 0.069 mm^{-1}). Data were collected with a colourless irregular block (0.42 × 0.31 × 0.25 mm). Of 23175 measured reflections (2θ: 3.6-54.0º), 4466 were unique (R_{int} = 0.0273). Final agreement factors were $R1$ = 0.0387 (3605 observed reflections) and $wR2$ = 0.1039. Data/restrains/parameters 4466/0/261; GOF = 1.021. Largest peak and hole in the final difference map 0.249 and -0.181 e Å^{-3}.

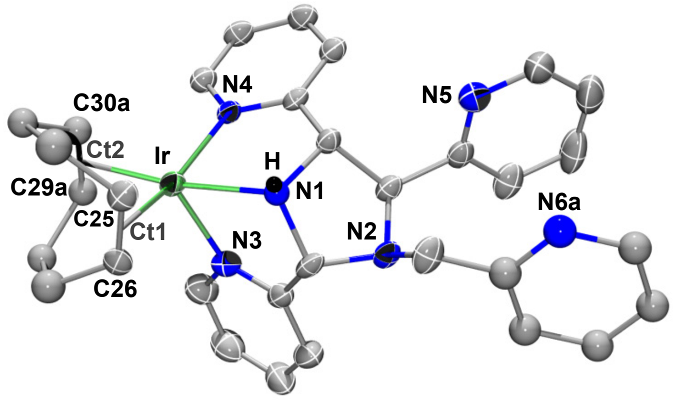

Figure S4. Molecular structure (ORTEP, ellipsoids set at 50% probability) of complex [4]Cl. Selected bond distances (Å) and angles (°): Ir–N1 2.136(4), Ir–N3 2.238(4), Ir–N4 2.119(4), Ir–Ct1 1.985(5), Ir–Ct2a 1.984(9), , Ir–Ct2b 1.982(14) N1–Ir–N4 79.3(2), N3–Ir-N4 85.6(2), N4–Ir–Ct1 160.7(2), N1–Ir–Ct2a 171.9(3), N1–Ir–Ct2b 157.2(4). Ct1, Ct2a and Ct2b are the middle points of C25-C26, C29a-C30a and C29b-C30b bonds, respectively.

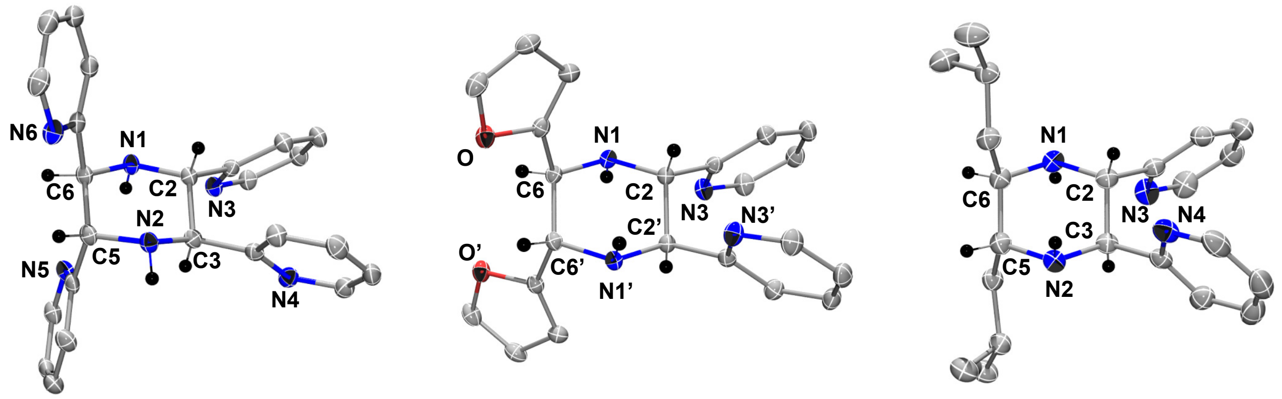

Figure S5. Molecular structure (ORTEP, ellipsoids set at 50% probability) of piperazines **6a** (left), **6d** (middle), and **6i** (right). Primed atoms are related to the unprimed ones by (1-x, 2-y, z).

NMR spectra of complexes, imines and piperazines

Complexes 3 and [4]Cl

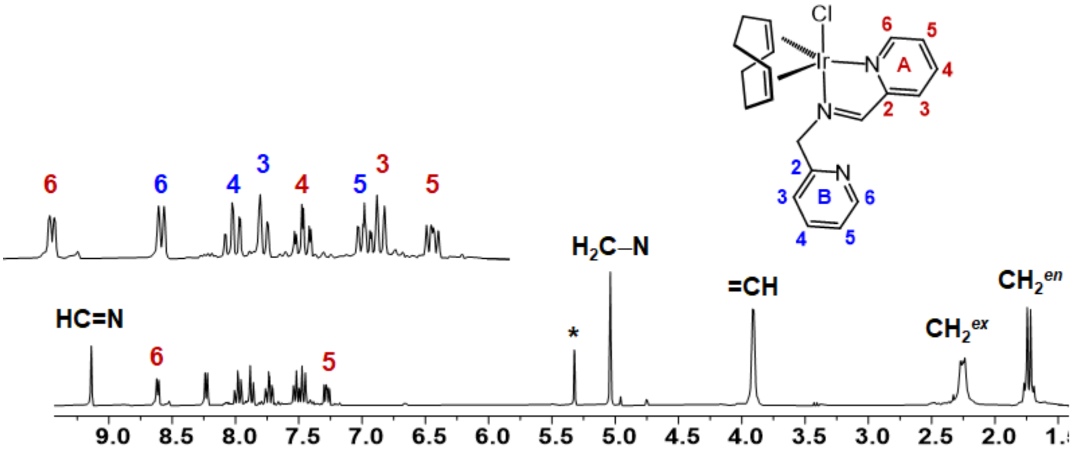

Figure S6. ^{1}H NMR spectrum of [IrCl(cod)(Py–CH=N–CH_2Py)] (**3**) in CD_2Cl_2 at –10 ºC. The asterisk (*) indicates the residual signal of the solvent.

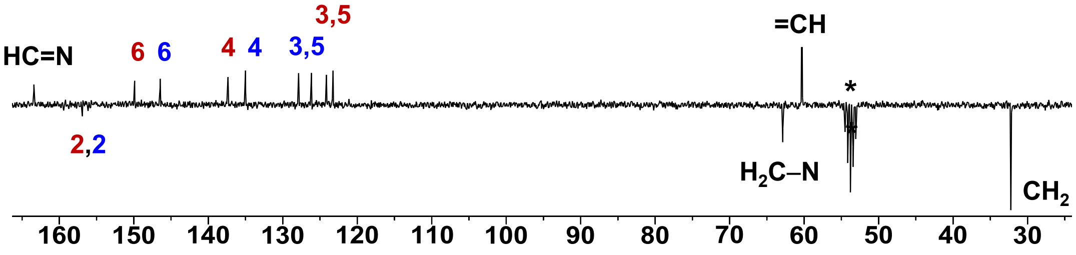

Figure S7. ^{13}C{^{1}H}-apt NMR spectrum of [IrCl(cod)(Py–CH=N–CH_2Py)] (**3**) in CD_2Cl_2 at –10 ºC. The asterisk (*) indicates the residual signal of the solvent.

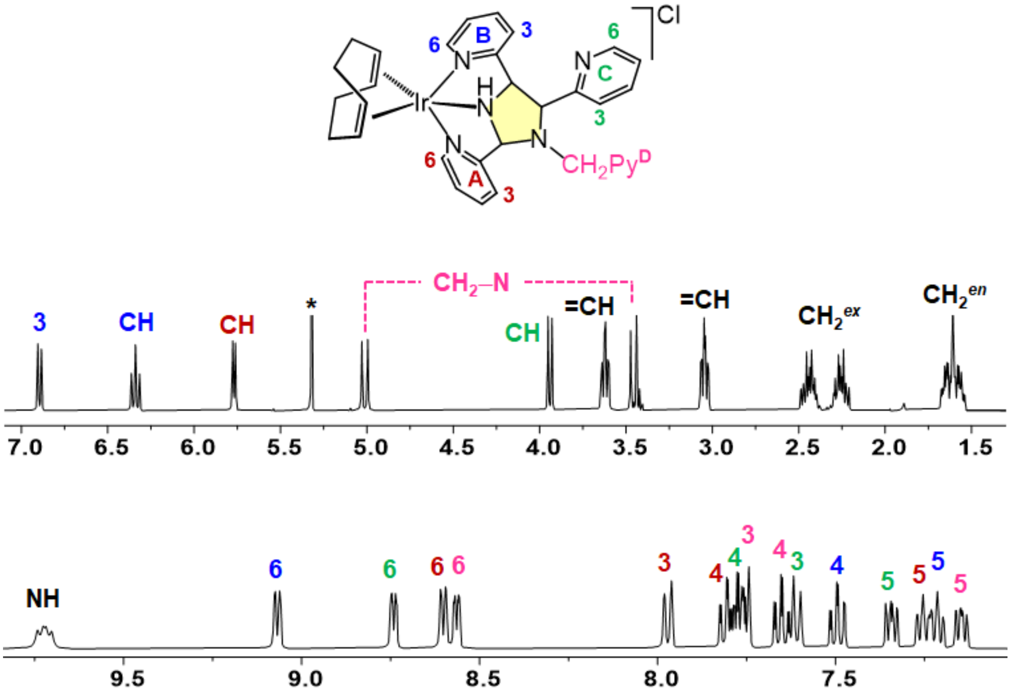

Figure S8. ^{1}H NMR spectrum of [Ir(cod)($C_{24}H_{22}N_6$)]Cl ([**4**]Cl) in CD_2Cl_2 in the range of 9.8 to 7.0 ppm (bottom) and from 7.0 to 1.5 ppm (top). The asterisk (*) indicates the residual signal of the solvent.

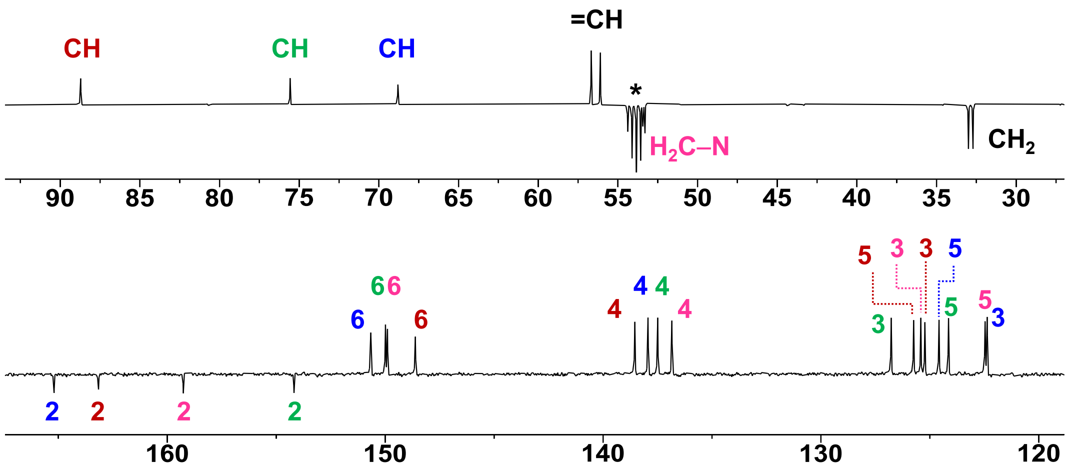

Figure S9. a) $^{13}C\{^1H\}$-apt NMR spectrum of $[Ir(cod)(C_{24}H_{22}N_6)]Cl$ [**4**]Cl in CD_2Cl_2 in the range of 168 to 118 ppm (bottom) and from 98 to 27 ppm (top). The asterisk (*) indicates the residual signal of the solvent.

Imines

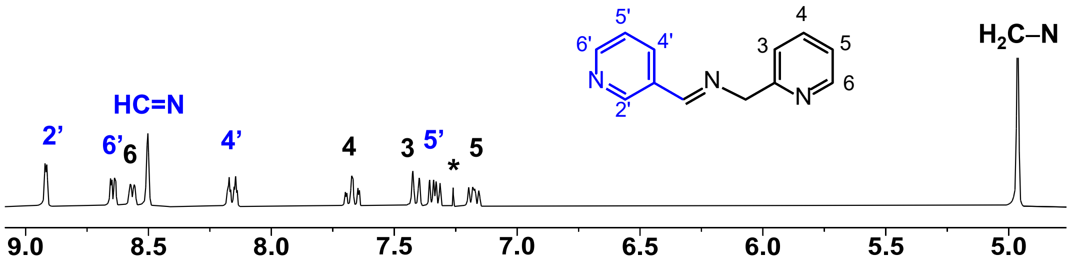

Figure S10. ^1H NMR spectrum of N-(pyridin-2-ylmethyl)-1-(pyridin-3-yl)methanimine (**2b**) in $CDCl_3$. The asterisk (*) indicates the residual signal of the solvent.

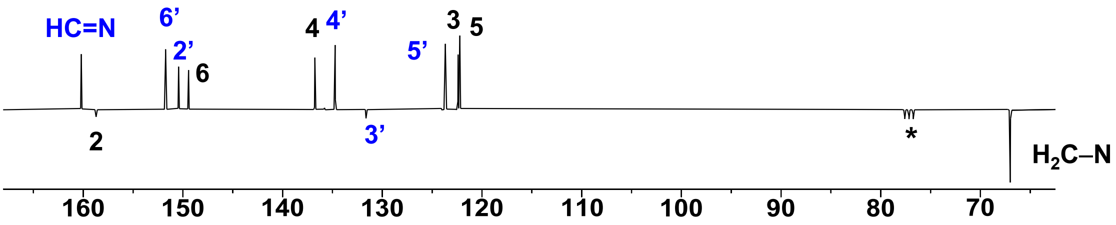

Figure S11. $^{13}C\{^1H\}$-apt NMR spectrum of N-(pyridin-2-ylmethyl)-1-(pyridin-3-yl)methanimine (**2b**) in $CDCl_3$. The asterisk (*) indicates the residual signal of the solvent.

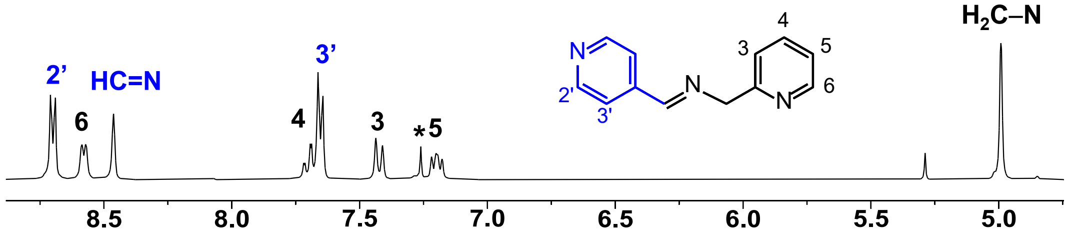

Figure S12. ^1H NMR spectrum of N-(pyridin-2-ylmethyl)-1-(pyridin-4-yl)methanimine (**2c**) in $CDCl_3$. The asterisk (*) indicates the residual signal of the solvent.

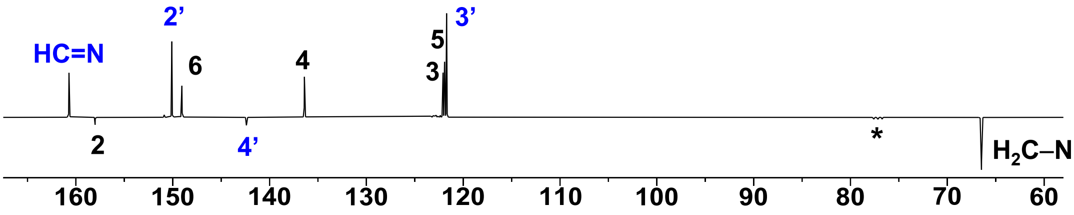

Figure S13. $^{13}C\{^{1}H\}$-apt NMR spectrum of N-(pyridin-2-ylmethyl)-1-(pyridin-4-yl)methanimine (**2c**) in $CDCl_3$. The asterisk (*) indicates the residual signal of the solvent.

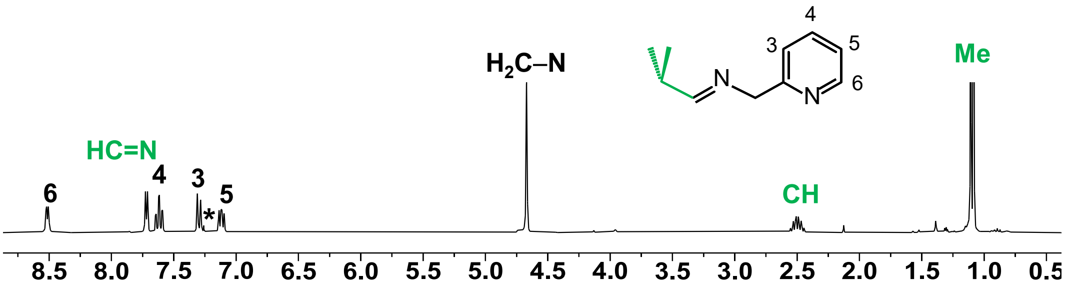

Figure S14. ^{1}H NMR spectrum of 2-methyl-N-(pyridin-2-ylmethyl)propan-1-imine (**2h**) in $CDCl_3$. The asterisk (*) indicates the residual signal of the solvent.

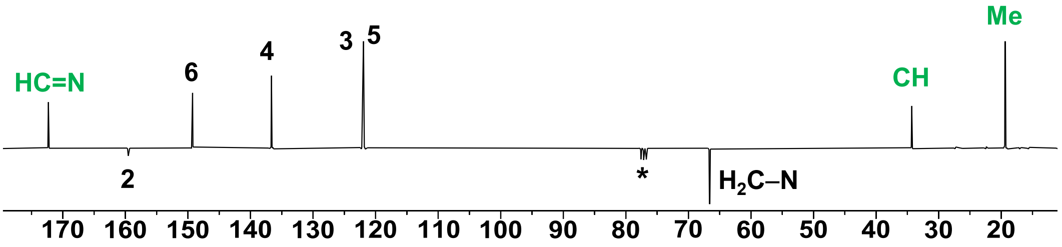

Figure S15. $^{13}C\{^{1}H\}$-apt NMR spectrum of 2-methyl-N-(pyridin-2-ylmethyl)propan-1-imine (**2h**) in $CDCl_3$. The asterisk (*) indicates the residual signal of the solvent.

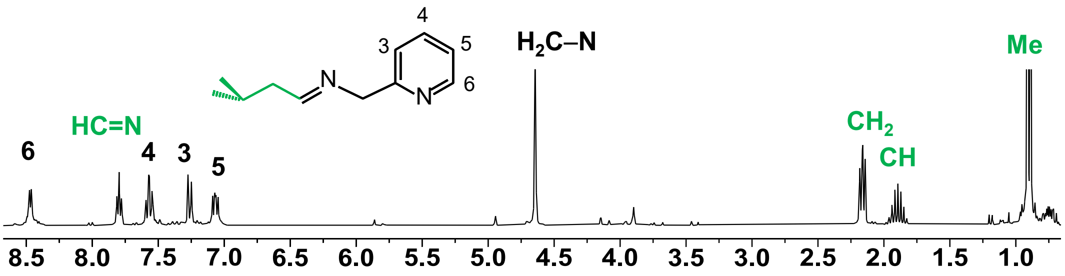

Figure S16. ^{1}H NMR spectrum of 3-methyl-N-(pyridin-2-ylmethyl)butan-1-imine (**2i**) in $CDCl_3$.

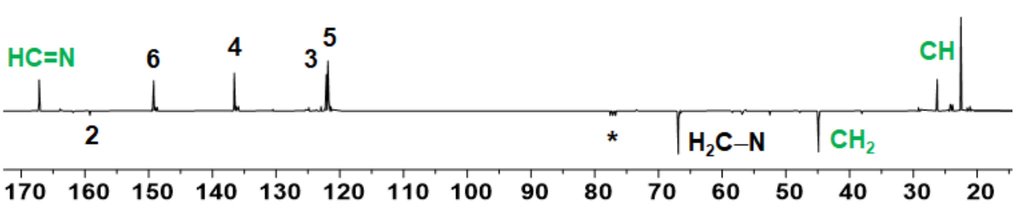

Figure S17. $^{13}C\{^{1}H\}$-apt NMR spectrum of 3-methyl-N-(pyridin-2-ylmethyl)butan-1-imine (**2i**) in $CDCl_3$. The asterisk (*) indicates the residual signal of the solvent.

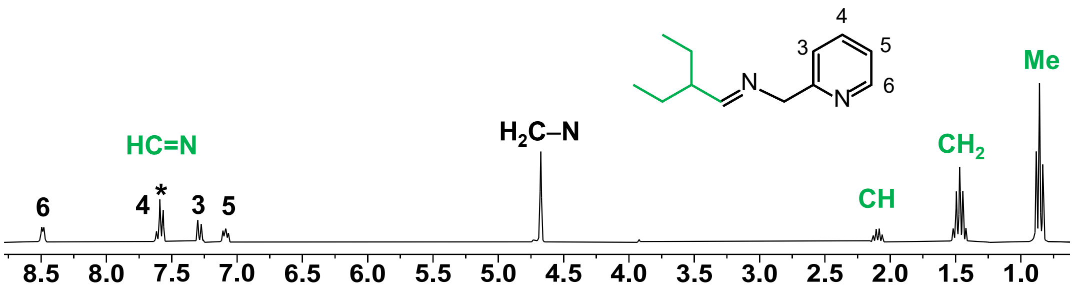

Figure S18. ^{1}H NMR spectrum of 2-ethyl-N-(pyridin-2-ylmethyl)butan-1-imine (**2j**) in $CDCl_3$. The asterisk (*) indicates the residual signal of the solvent.

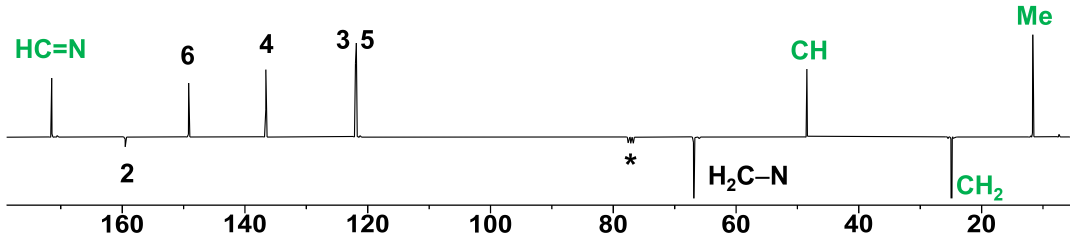

Figure S19. ^{13}C{^{1}H}-apt NMR spectrum of 2-ethyl-N-(pyridin-2-ylmethyl)butan-1-imine (**2j**) in $CDCl_3$. The asterisk (*) indicates the residual signal of the solvent.

Piperazines

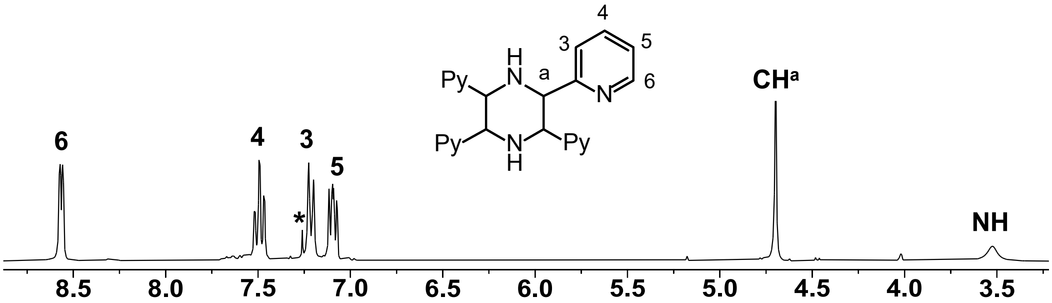

Figure S20. ^{1}H NMR spectrum of 2,3,5,6-tetra(pyridin-2-yl)piperazine (**6a**) in $CDCl_3$. The asterisk (*) indicates the residual signal of the solvent.

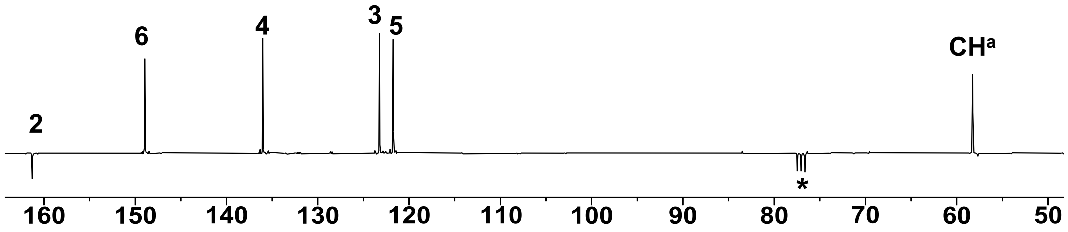

Figure S21. ^{13}C{^{1}H}-apt NMR spectrum of 2,3,5,6-tetra(pyridin-2-yl)piperazine (**6a**) in $CDCl_3$. The asterisk (*) indicates the residual signal of the solvent.

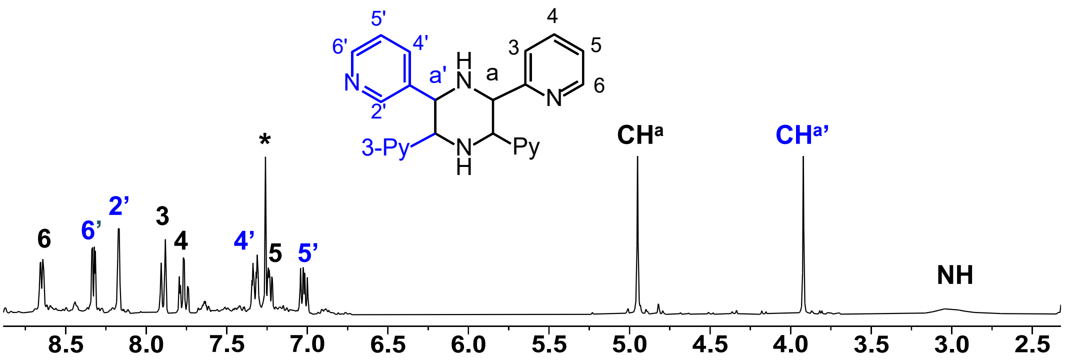

Figure S22. ^{1}H NMR spectrum of 2,3-di(pyridin-2-yl)-5,6-di(pyridin-3-yl)piperazine (**6b**) in $CDCl_3$. The asterisk (*) indicates the residual signal of the solvent.

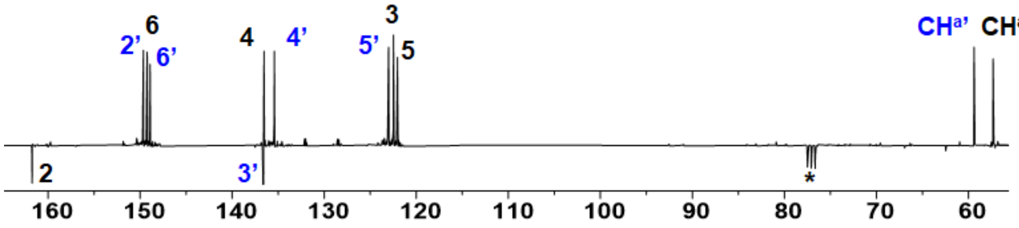

Figure S23. ^{13}C{^{1}H}-apt NMR spectrum of 2,3-di(pyridin-2-yl)-5,6-di(pyridin-3-yl)piperazine (**6b**) in $CDCl_3$. The asterisk (*) indicates the residual signal of the solvent.

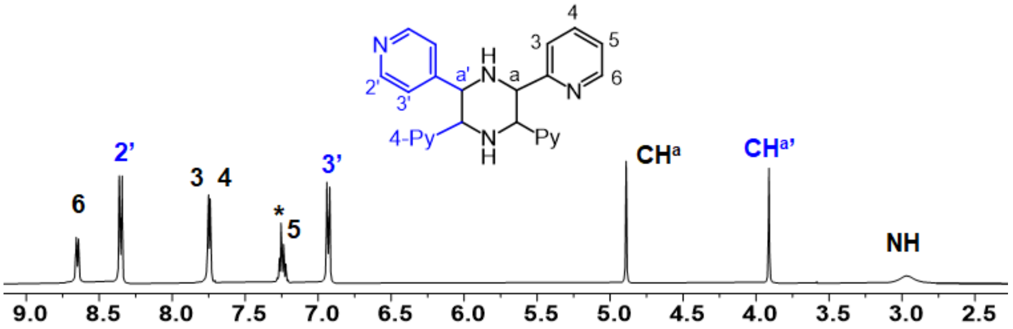

Figure S24. ^{1}H NMR spectrum of 2,3-di(pyridin-2-yl)-5,6-di(pyridin-4-yl)piperazine (**6c**) in $CDCl_3$. The asterisk (*) indicates the residual signal of the solvent.

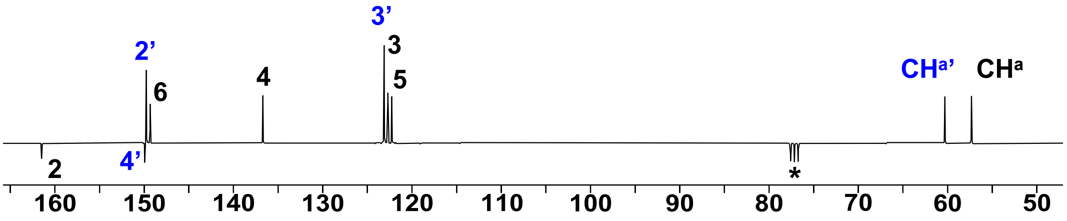

Figure S25. ^{13}C{^{1}H}-apt NMR spectrum of 2,3-di(pyridin-2-yl)-5,6-di(pyridin-4-yl)piperazine (**6c**) in $CDCl_3$. The asterisk (*) indicates the residual signal of the solvent.

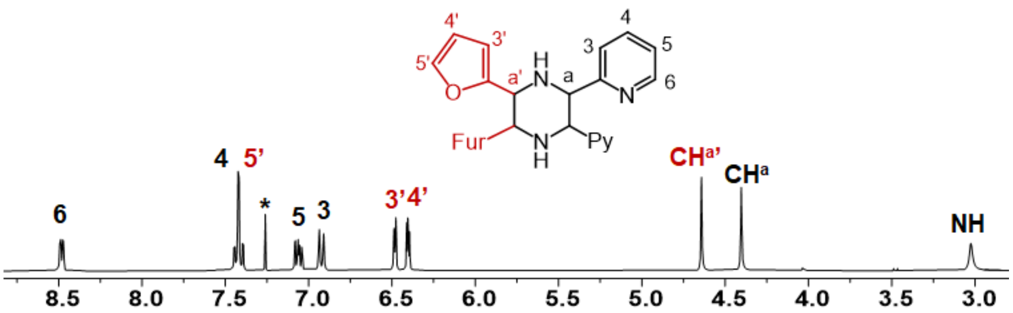

Figure S26. ^{1}H NMR spectrum of 2,3-di(furan-2-yl)-5,6-di(pyridin-2-yl)piperazine (**6d**) in $CDCl_3$. The asterisk (*) indicates the residual signal of the solvent.

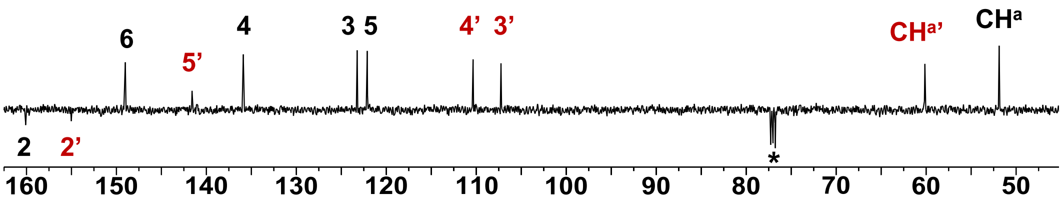

Figure S27. ^{13}C{^{1}H}-apt NMR spectrum of 2,3-di(furan-2-yl)-5,6-di(pyridin-2-yl)piperazine (**6d**) in $CDCl_3$. The asterisk (*) indicates the residual signal of the solvent.

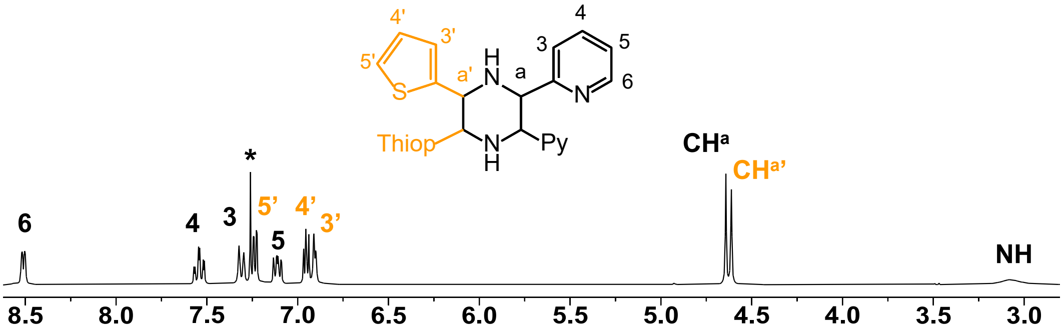

Figure S28. ^{1}H NMR spectrum of 2,3-di(pyridin-2-yl)-5,6-di(thiophen-2-yl)piperazine (**6e**) in $CDCl_3$. The asterisk (*) indicates the residual signal of the solvent.

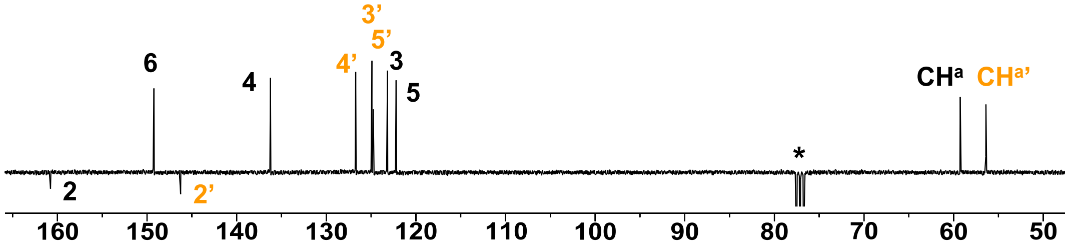

Figure S29. ^{13}C{^{1}H}-apt NMR spectrum of 2,3-di(pyridin-2-yl)-5,6-di(thiophen-2-yl)piperazine (**6e**) in $CDCl_3$. The asterisk (*) indicates the residual signal of the solvent.

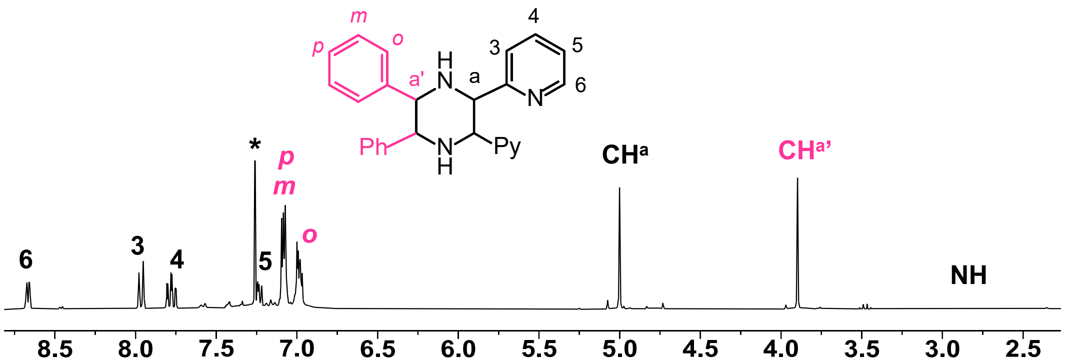

Figure S30. ^{1}H NMR spectrum of 2,3-diphenyl-5,6-di(pyridin-2-yl)piperazine (**6g**) in $CDCl_3$. The asterisk (*) indicates the residual signal of the solvent.

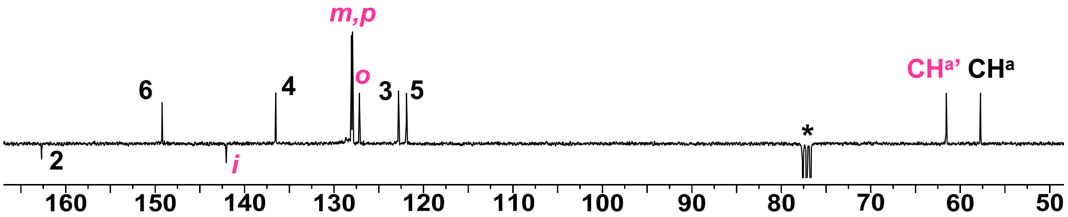

Figure S31. ^{13}C{^{1}H}-apt NMR spectrum of 2,3-diphenyl-5,6-di(pyridin-2-yl)piperazine (**6g**) in $CDCl_3$. The asterisk (*) indicates the residual signal of the solvent.

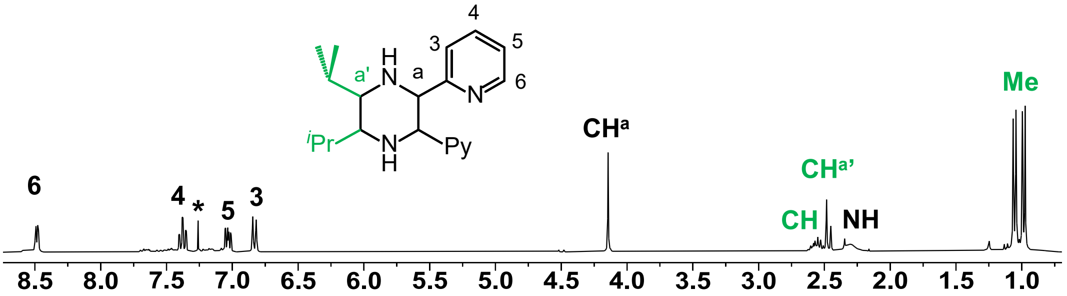

Figure S32. ^{1}H NMR spectrum of 2,3-diisopropyl-5,6-di(pyridin-2-yl)piperazine (**6h**) in $CDCl_3$. The asterisk (*) indicates the residual signal of the solvent.

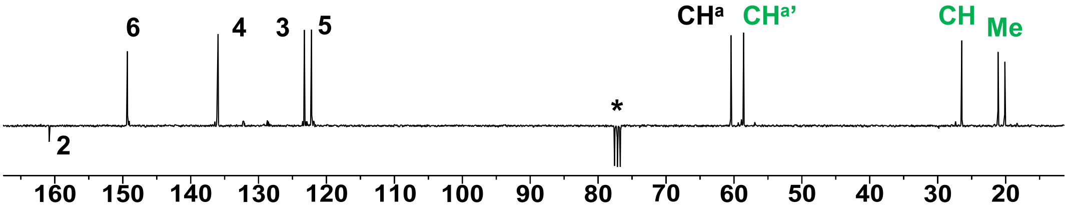

Figure S33. ^{13}C{^{1}H}-apt NMR spectrum of 2,3-diisopropyl-5,6-di(pyridin-2-yl)piperazine (**6h**) in $CDCl_3$. The asterisk (*) indicates the residual signal of the solvent.

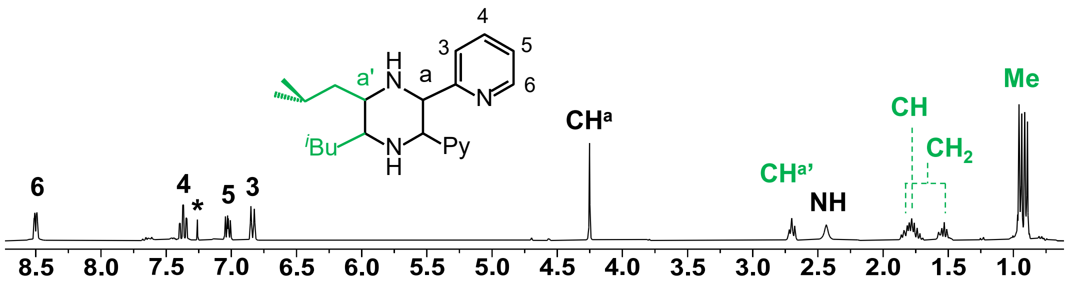

Figure S34. ^{1}H NMR spectrum of 2,3-diisobutyl-5,6-di(pyridin-2-yl)piperazine (**6i**) in $CDCl_3$. The asterisk (*) indicates the residual signal of the solvent.

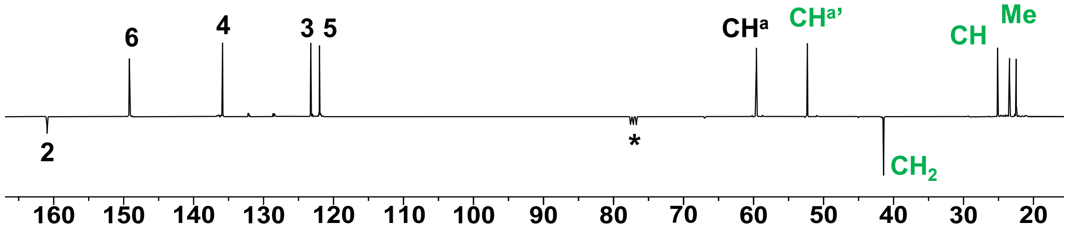

Figure S35. ^{13}C{^{1}H}-apt NMR spectrum of 2,3-diisobutyl-5,6-di(pyridin-2-yl)piperazine (**6i**) in $CDCl_3$. The asterisk (*) indicates the residual signal of the solvent.

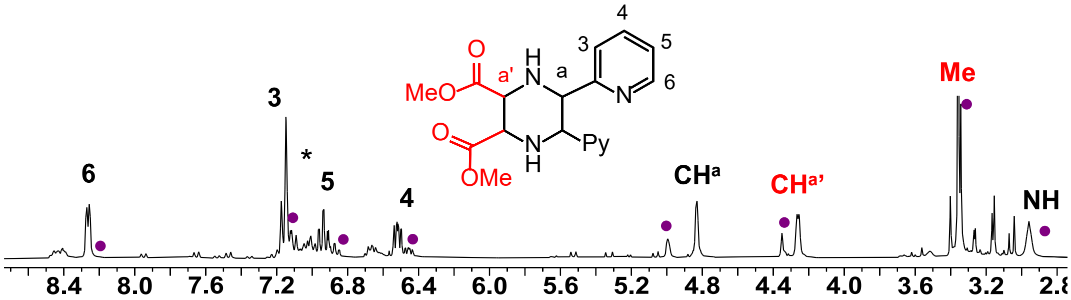

Figure S36. ^{1}H NMR spectrum of dimethyl 5,6-di(pyridin-2-yl)piperazine-2,3-dicarboxylate (**6m**) in C_6D_6. The asterisk (*) indicates the residual signal of the solvent. The purple circle indicates the minor isomer (**6m'**).

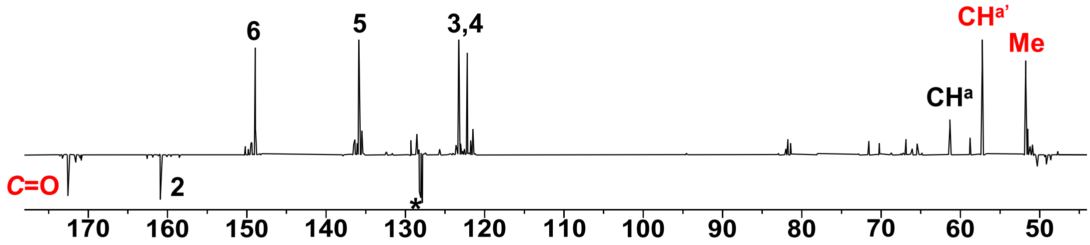

Figure S37. ^{13}C{^{1}H}-apt NMR spectrum of dimethyl 5,6-di(pyridin-2-yl)piperazine-2,3-dicarboxylate (**6m**) in C_6D_6. The asterisk (*) indicates the residual signal of the solvent.

References

(S1) Herde, J. L.; Lambert, J. C.; Senoff, C. V.; Cushing, M. A. Cyclooctene and 1,5-Cyclooctadiene Complexes of Iridium(I). *Inorg. Synth.* **1974**, *15*, 18–20.

(S2) Crabtree, R. H.; Morris, G. E. Some Diolefin Complexes of Iridium(I) and a Trans-Influence Series for the Complexes [IrCl(cod)L]. *J. Organomet. Chem.* **1977**, *135*, 395–403.

(S3) D'Hollander, A. C. A.; Romero, E.; Vijayakumar, K.; Le Houérou, C.; Retailleau, P.; Dodd, R. H.; Iorga, B. I.; Cariou, K. Base-Mediated Generation of Ketenimines from Ynamides: [3+2] Annulation with Azaallyl Anions. *Adv. Synth. Catal.* **2021**, *363*, 2903–2908.

(S4) Padilla, S.; Tejero, R.; Adrio, J.; Carretero, J. C. *N* -(2-Pyridylmethyl)Imines as Azomethine Precursors in Catalytic Asymmetric [3 + 2] Cycloadditions. *Org. Lett.* **2010**, *12*, 5608–5611.

(S5) Martínez-Martínez, D.; Santiago, M. L.; Toscano, R. A.; Amézquita-Valencia, M. Molybdenum (VI) Complexes Containing Pyridylimine Ligands: Effect of the Imine Nitrogen Substituent in the Epoxidation Reaction. *Eur. J. Inorg. Chem.* **2021**, 243–251.

(S6) Schoumacker, S.; Hamelin, O.; Téti, S.; Pécaut, J.; Fontecave, M. Activation of Oxaziridines by Lewis Acids: Application in Enantioselective Sulfoxidation. *J. Org. Chem.* **2005**, *70*, 301–308.

(S7) de Bruin, B.; Kicken, R. J. N. A. M.; Suos, N. F. A.; Donners, M. P. J.; den Reijer, C. J.; Sandee, A. J.; de Gelder, R.; Smits, J. M. M.; Gal, A. W.; Spek, A. L. Diversity in Complexation of $[Rh^{I}(cod)]^{+}$ and $[Ir^{I}(cod)]^{+}$ by Pyridine-Amine-Pyrrole Ligands. *Eur. J. Inorg. Chem.* **1999**, 1581–1592.

(S8) Yang, H.; Lu, S. N.; Chen, Z.; Wu, X.-F. Silver-Mediated [3 + 2] Cycloaddition of Azomethine Ylides with Trifluoroacetimidoyl Chlorides for the Synthesis of 5-(Trifluoromethyl)imidazoles. *J. Org. Chem.* **2021**, *86*, 4361–4370.

(S9) Sheldrick, G. M. SADABS, Bruker AXS, Madison, WI (USA), 1997.

(S10) Sheldrick, G. M. *Acta Cryst.* **2008**, *A64*, 112–122.

(S11) Sheldrick, G. M. *Acta Cryst.* **2015**, *C71*, 3–8.

(S12) Farrugia, L. J. *J. Appl. Crystallogr.* **2012**, *45*, 849–854.

(S13) Spek, A. L. *Acta Cryst.* **2015**, *C71*, 9–18.
